# Drug-Related Problems Identified During Pharmacy Intervention and Consultation: Implementation of an Intensive Care Unit Pharmaceutical Care Model

**DOI:** 10.3389/fphar.2020.571906

**Published:** 2020-09-11

**Authors:** Xiao-xiao Li, Si-qian Zheng, Jia-hui Gu, Tao Huang, Fang Liu, Qing-gang Ge, Bin Liu, Chao Li, Min Yi, You-fa Qin, Rong-sheng Zhao, Lu-wen Shi

**Affiliations:** ^1^ Department of Pharmacy, Peking University Third Hospital, Beijing, China; ^2^ Department of Pharmacy Administration and Clinical Pharmacy, School of Pharmaceutical Sciences, Peking University, Beijing, China; ^3^ International Research Center for Medicinal Administration, Peking University, Beijing, China; ^4^ Department of Intensive Care Unit, Peking University Third Hospital, Beijing, China; ^5^ Information Management and Big Data Center, Peking University Third Hospital, Beijing, China; ^6^ Department of Clinical Pharmacy, SSL Center Hospital of Dongguan City, Dongguan, China

**Keywords:** critical care, hospital medicine, pharmacists, patient safety, medical error

## Abstract

**Aim:**

To identify common drug-related problems (DRPs) during pharmacy intervention and consultation in an intensive care unit (ICU); to explore the gap between physicians and pharmacists on their understanding of each other’s capabilities and needs.

**Method:**

We conducted a single-center prospective study in the ICU of a tertiary academic hospital for 21 months. A pharmaceutical care (PC) model was implemented by a pharmacy team, and data were collected during pharmacy intervention and consultation. Data analysis was performed on identified DRPs, causes and their relationships. DRPs’ frequency during intervention and consultation was compared. Problem-level descriptive analysis and network analysis were conducted using R 3.6.3.

**Result:**

Implementation of PC model greatly improved the efficacy of pharmacists in both interventions proposed to solve DRPs (from 13.6 to 20.1 cases per month) and number of patients being closely monitored (from 7.7 to 16.9 per month). Pharmacists identified 427 DRPs during pharmacy intervention with primarily adverse drug events (ADEs, 34.7%) and effect of treatment not optimal (25.5%), and 245 DRPs during consultation (mainly ADEs, 58.4%). About three-fifths DRPs were caused by antibiotics. Comparing DRPs identified during pharmacy intervention and consultation, physicians consulted pharmacists more on questions related to medication safety, while pharmacists also paid attention to treatment effectiveness, which was consulted less commonly.

**Conclusion:**

Implementation of PC model is beneficial in guiding pharmacy practice and improving efficacy especially under limited human resources. Physicians and pharmacists shall continue ensuring drug safety and be familiar with the scope of PC and clinical need for a better cooperation.

## Highlights

*What is already known about this subject*

- Critical care pharmacists can effectively and efficiently offer specialized recommendations in complex pharmacotherapies.
- Imbalance exists in the development of pharmaceutical care among different specialties and various regions at present.
- A unified standard for ICU pharmaceutical care practice has yet mapped out in China.

*What this study adds*

- The implementation of an ICU pharmaceutical care model can guide clinical practice and potential enhance the overall efficacy.
- Treatment safety was the most frequent drug-related problems identified during pharmacy intervention and consultation.
- Physicians and pharmacist should gain a better understanding of each other by learning the scope of pharmaceutical care and clinical requirements.

## Introduction

Since the publication of the report *Harvard Medical Practice study* (Brennan et al., 1991; Leape et al., 1991), drug-related problems (DRPs) that might lead to adverse drug events (ADEs) have received extensive attention among the public and health care system worldwide. The observed rate of DRP was about 5.6 per 100 patient admissions, with almost half of DRPs being potentially preventable (Leendertse et al., 2008). DRPs also place a substantial health and economic burden on patients and the health care system, which cost €2.58 to €111 727.08 per medication error (Walsh et al., 2017). Patients admitted to the intensive care unit (ICU) are at higher risk for developing DRPs, from 2.1/1,000 to 804.5/1,000 patients days (Garrouste-Orgeas et al., 2010; Garrouste-Orgeas et al., 2015; Garrouste-Orgeas et al., 2016), primarily cause by their critical diseases status or complications, the use of various high alert medications, and the rapid changing pharmacotherapies (Kari et al., 2018).

Previous studies have shown that critical care pharmacists can play an essential role in promoting the delivery of pharmaceutical care (PC) and improve the overall quality of health care by offering individualized recommendations in complicated drug regimens, reducing the incidence rate of DRPs, and decreasing preventable ADEs (Tasaka et al., 2018; Lee et al., 2019; Reinau et al., 2019). However, the development of PC in the ICU is currently facing three major challenges worldwide. Firstly, only a few guidelines provided recommendations on delivery of PC to critically ill patients. A position paper described fundamental, desirable, and optimal pharmacy services and requirements for relative personnel from a broad perspective (Rudis and Brandl, 2000); however, concrete guidelines with details on high-risk patient identification and therapeutic monitoring are needed to guide pharmacists’ daily practice. Secondly, imbalance exists in the development of PC among different specialties and various regions (Lee et al., 2019). Last but not the least, while the pharmacy profession is widely recognized, and pharmacists have become an essential member of the multidisciplinary team (MDT) (Leape et al., 1999; Zhou et al., 2019), a gap still exists between physicians and pharmacists on their understanding of each other’s capabilities and needs. It forms a virtual barrier, and prevents both sides from forming a deep cooperative relationship, even in areas with developed PC system.

The ICU PC development situation in China is further complicated by the relatively late introduction of the concept and limited human resources. Being first advocated in the United States in 1950s (Anderson, 1992), the concept of PC and clinical pharmacy was not introduced to China until 1990s (Hu et al., 2014). Research in this area is scarce, and only a few studies have been published to discuss DRPs. Moreover, while the United States has 14.9 hospital pharmacists available per 100 hospital beds (Schneider et al., 2019), the number was estimated to be 1.4 to 2.4 in China (Li et al., 2019). Limited human resources made it difficult for pharmacists to provide a comprehensive and daily on-ward participation of MDT. Instead, some clinical pharmacists could only focus on off-ward services, such as therapeutic drug monitoring (TDM), on-call duty for consultations, reevaluation of the prescriptions, and pharmaceutical information service (Wei et al., 2011; Penm et al., 2014b). Thus, a standard pharmacy practice model is needed to promote the development of PC in the ICU setting, and a discussion on the classifications and incidence of DRPs is necessary to guide the future efforts in reducing the incidence of DRPs.

We therefore developed a PC model that was tailored to our surgical intensive care unit (SICU) setting, and conducted a prospective study to explore the following questions: (1) Can this PC model guide pharmacists’ daily practice properly and help pharmacists identify patients in greater need of PC under limited human resource? Would it potentially improve the efficacy of ICU pharmacists? (2) What are the most common DRPs in the ICU and what are the causes? (3) Is there any difference of DRPs identified during pharmacy intervention (offered by pharmacists) and consultation (requested by physicians)? We hope that the answer to this question can help us achieve a better understanding of physicians’ and pharmacists’ needs and capabilities, and provide a new angle for deepening their bilateral cooperative relationship.

## Methods

### Setting and Study Design

This is a single-center prospective study conducted in the SICU of a 1891-bed class A tertiary academic hospital, located in Beijing, China, with a duration of 21-month from January, 2018 to September, 2019. The SICU had 19 open beds to admit primarily patients within the hospital for perioperative management, caring for 101 patients per month, with average case-mix index of 4.2 and an overall mortality of 3.8%. One patient on the unit had five to six medication existing orders per day including three to four new orders. Three quarters of the patients were from general surgery, urology, gynecology and obstetrics, orthopedic, and neurosurgery departments. This study was approved by the ethical review board of Peking University Health Science Center, and informed consent of participant was exempted (IRB00001052-20014).

### Pharmaceutical Care Services Provided in SICU

#### The Content of SICU Pharmaceutical Care Model

In this study, the pharmacy team used a newly developed PC model to guide pharmacy practice. The pharmacy team was consisted of one leading pharmacist, a master student in clinical pharmacy major, and/or a pharmacist on clinical pharmacy training. The PC model, as shown in **Table 1**, defined high-risk patient populations and classes that need priority monitoring, with an additional list of pharmacy services that should be provided to ensure medication safety. Critical care guidelines and books (Ministry of Health, 2004; Kellum et al., 2013; Ministry of Health, 2015; Taskforce et al., 2015; Chou et al., 2016; Frontera et al., 2016; McClave et al., 2016; Ye et al., 2016; Rhodes et al., 2017; Borthwick et al., 2018; Liu et al., 2018) were consulted to set up the framework of this model. Then, the pharmacy team finalized patient populations and drug classes that need priority monitoring after reviewing past experience of 1.5 years on the unit and having several rounds of discussion with the physicians (LC, GQG). Additionally, details and key points were added for each patient population, drug class, and pharmacy services listed to guide the pharmacy practice. Two deputy directors of the SICU department (GQG, YM) and one senior pharmacist (LF) were involved to supervise the implementation of the PC model.

**Table 1:** Pharmaceutical care model for the surgical intensive care unit.

Pharmacy services			Key Points	
**Patient population that need close monitoring**				
**Severe comorbidities**	Renal insufficiency (without renal replacement therapy)		Evaluate the degree and causes of renal insufficiency, select appropriate drugs, and give recommendations for dose adjustments. Monitor creatinine clearance, urea nitrogen, and urine output.	
	Liver insufficiency		Evaluate the type and degree of liver dysfunction, select appropriate drugs, and give recommendations for dose adjustment. Monitor bio-chemical indicators such as transaminase and bilirubin.	
	Other chronic diseases		Evaluate the appropriateness of drug selection, and give recommendations for dose adjustments of certain medications such as antidiabetic agents, antihypertensive drugs. Monitor body temperature, heart rate, breath, blood pressure, blood sugar, changes in intake and output volume, etc.	
**Special Populations**	Hemodynamic instability		Evaluate patient’s hemodynamic condition. Select appropriate vasoactive drugs and give recommendations on dose adjustments.	
	Severe infections		Guide the correct collection of specimens, interpret drug sensitivity results, and provide anti-infective therapy recommendations.	
	Renal replacement therapy (RRT)		Adjust dose according to the mode of RRT and pharmacokinetic characteristics of the medications, such as clearance pathway, protein binding rate, apparent distribution volume, molecular weight, etc.	
	Perinatal period		Evaluate the appropriateness of drug selection (consider drug distribution in peritoneal area and placental barrier).	
	Obesity		Adjust dose according to drug characteristics.	
	History of drug or food allergy		Avoid contact with allergens and provide alternative solutions.	
**Drugs that need close monitoring**				
**Nutrition therapy**			Conduct nutritional risk screening for inpatients and provide nutritional support recommendations for patients who are in need ; For patients need parenteral nutrition: Evaluate the appropriateness of timing, infusion route (central vein or peripheral vein), and the rationality of nutritional components. Monitor mechanical complications (pneumothorax, catheter embolism, phlebitis, etc.), infection complications (commonly seen in central venous catheter), metabolic complications (monitor blood glucose, lipid level, and electrolytes such as potassium, sodium, blood calcium, phosphorus, magnesium, etc.). For patients need enteral nutrition: Evaluate the appropriateness of timing, enteral nutrition route (nasogastric tube, nasal empty tube, gastrostomy and jejunostomy), and assist the selection of enteral nutrition preparation and the feeding volume. Monitor if patient can tolerate the therapy (such as nausea, vomiting, diarrhea, etc.), signs and symptoms (especially abdominal pain, bloating, bowel sounds, etc.) of intolerance, and excretions (urine, stool). Be cautious on inhalation and aspiration pneumonia.	
**Antibiotics**			Assess if the patient has real infection, Evaluate the possible infected area and the pathogenic bacteria according to patient’s clinical manifestations (consciousness, heart rate, body temperature, intake and output volume, sputum and urine characteristics, etc.), lab values (leukocytes, neutrophils, procalcitonin, albumin, hemoglobin, etc.), imaging and etiology examination. Choose appropriate antibiotic drugs and give individual dose adjustments if necessary (consider the infectious site, severity of the infection, renal function, weight and age, etc.). Monitor anti-infective therapy effects and possible adverse reactions, and change antibiotics if necessary.	
**Anticoagulant drug**			Pay attention to the bridging, and monitor bleeding and drug interaction of patients who require long-term use of warfarin, new oral anticoagulants and other drugs for routine anticoagulation due to atrial fibrillation, percutaneous coronary intervention (PCI), valve replacement, etc. For patients on anticoagulants to prevent or treat deep vein thrombosis, pulmonary embolism, and patients need perioperative anticoagulation, pay attention to the treatment course and dosage of anticoagulant therapy, and monitor changes in patient’s coagulation function and renal function.	
**Drugs on the Key Monitoring list of the hospital**			Cautiously assess the indications, usage and dosage, treatment courses of patients on such medications to ensure appropriate use.	
**Drugs with special dosage forms**			If nasal feeding needs to destroy the original dosage form (enteric-coated tablets, sustained-release tablets, control tablets, etc.), evaluate whether it is appropriate and the need of replacement with plain tablets.	
**Other drugs**			Pay attention to the selection of antiepileptic drugs and liver protecting drugs.	
**Pharmaceutical care services**				
**Medication order review**			Drug selection (including drugs, dosage forms, solvents, etc.), dosing, and frequency adjustments.	
**Individualized drug dosing regimen based on therapeutic drug monitoring (TDM) results**			Interpret the TDM results comprehensively; design or optimize the drug regimen accordingly based on TDM results and other clinical information.	
**Identify adverse drug reactions**			Analyze medical records and use package inserts or relevant evidence to identify the drug that most likely to cause the reported adverse reaction. Report adverse drug reactions. Make recommendations for drug replacement.	
**Identify potential drug-drug interactions (DDI)**			Identify drugs with potential DDI, and make interventions including avoid use, replacement, dose adjustment, monitoring and remind physicians. Pay extra attention to drugs with the same adverse reactions, CYP450 substrates/inducers/inhibitors, P-glycoprotein pump substrates/inducers/inhibitors, etc.	
**Pharmacy information**			Provide proper replies to the consults raised by medical staff in a timely manner.	

In the PC model, high-risk patient populations mainly included patients with baseline diseases before surgery (e.g., renal insufficiency), perioperative patients who had acute events (e.g., hemodynamic instability), and the special patient populations (e.g., obesity or pregnancy). Medications that need priority monitoring primarily included commonly used medications in perioperative patients (e.g., parenteral and enteral nutrition, antibiotics), medications that were recently marketed in China (e.g., novel oral anticoagulants) and medications that need therapeutic drug monitoring (e.g., vancomycin). The content of pharmacy services mainly included prescription review, medication safety ensurance, and drug information support.

#### SICU Pharmaceutical Care Model Implementation

During the study period, the care team used the PC model as a guide to identify patients and medications which need prioritizing pharmaceutical monitoring. For example, if a patient has reduced renal function, the severity and cause of renal dysfunction should be assessed by the PC team. Then, the medication regimen should be reviewed daily and dose adjustment recommendations should be made if needed; close monitoring of the patient’s fluid balance and serum creatinine clearance should also be performed. For patients on antibacterial medications, infection should first be confirmed by verifying patient clinical symptoms, lab values, together with results of imaging and etiological examinations. Moreover, drug regimen should be evaluated for safety and effectiveness.

On a daily basis, the PC team attended shift meeting and rounding in the morning, and spent 2.5 to 3.5 h a day on average at the SICU unit. The pharmacists checked new prescription orders of the patients mentioned in the shift meeting and the team they rounded with, and made face-to-face communications if any change needs to be made. For the rest of the time (4.5–5.5 h a day on average), the care team based at the pharmacy department, and communications were made through phone calls or a social software called *WeChat*. The care team also attended the weekly case discussion scheduled on every Wednesday. It usually covers one to two complicated patient cases, including dead cases if applicable.

### Data Collection and Classification

Data was collected by the leading pharmacist after providing pharmacy interventions and completing consultations. The information collected mainly includes case number, patient gender, description of the DRP, pharmacists’ recommendations, the consultation questions and answers, etc.

During DRP classification, the Pharmaceutical Care Network Europe (PCNE) Classification system was initially chosen for it considered as a validated system for DRP classification in hospital settings (Griese-Mammen et al., 2018; Qu et al., 2019). However, during the pilot study, we found that certain DRPs and their related causes were not included in the system. We therefore added a few columns and used a modified PCNE V9.0 Classification system for DRP analysis after reaching a consensus among all three members participated in this process, see **Supplementary Table 1**. For example, P3.3 was added for need additional drug therapy monitoring, and C1.8 was added for necessary genetic testing before drug initiation (for drugs such as carbamazepine). The classification system for pharmacy consultations was also created based on the original PCNE V9.0 Classification system using a similar strategy with pharmacy intervention classification.

**Figure 1:**
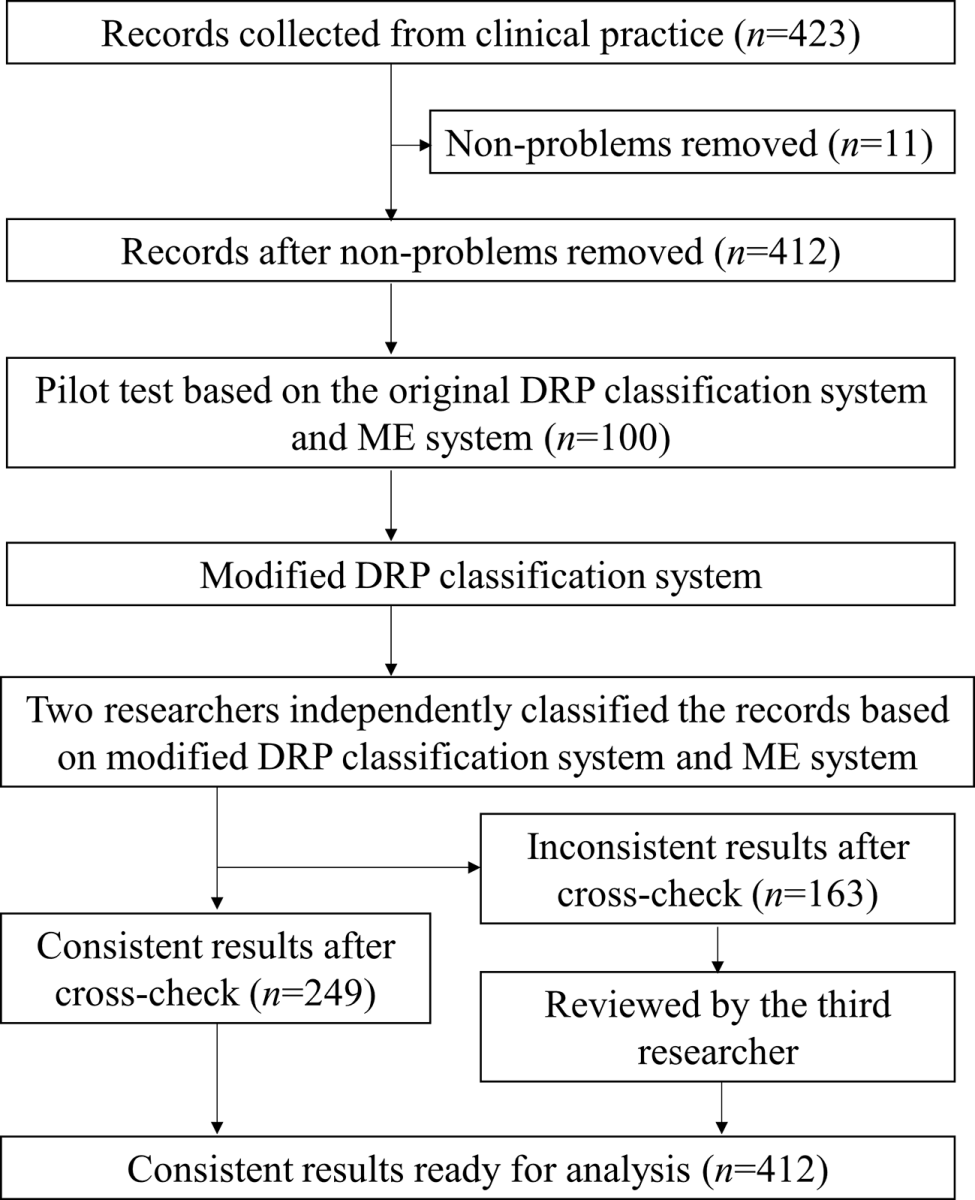
The flowchart for drug-related problems classification during pharmacy intervention. A flowchart was used to show the process of DRP classification using modified DRP classification system. Records removed (*n* = 11) were recommendations related to documentations or non-clinical issues.

During DRP classification, as shown in **Figure 1**, a pilot test was performed and DRPs collected were classified by two researchers (ZSQ, GJH) independently using the modified PCNE V9.0 classification system. A third researcher (LXX) made the final decision if classification results unmatched. The consultation was classified using the same method (see **Supplementary Figure 1**). In addition, the classification of medication errors (ME) was conducted by three researchers (ZSQ, QYF, LXX) using the National Coordinating Council for Medication Error Reporting and Prevention (NCC-MERP) classification criteria for pharmacy intervention (Brixey et al., 2002).

### Statistical Analysis

Problem-level descriptive analysis was used to characterize the DRPs and relevant causes and interventions. Monthly intervention rate and monthly average number of interventions per patient were calculated. Network analysis was performed to find the potential causes led to specific drug-related problems during pharmacy intervention and consultation separately. Additionally, DRPs identified during pharmacy intervention and consultation were compared in the purpose of discovering similarities and differences of the focus on medication use from the perspective of physicians and pharmacists. Categorical variables were described using frequency counts and percentages, and continuous variables were described using medians with interquartile range (IQR). All the calculation and analysis were conducted using R 3.6.3.

## Results

### Basic Information and Efficacy Evaluation

During the 21-month study, the pharmacy team performed close monitoring to a total number of 354 patients identified by using SICU PC model. The average age of the patient population was 57 years old (IQR 41, 76) and 56.8% of them were male (*n*=201). According to the model, these patients need priority monitoring as they had baseline diseases of renal dysfunction (including patients on renal replacement therapy; 19.2%, *n*=68), reduced heart function or blood pressure instability (11.3%, *n*=40), coagulopathy (10.5%, *n*=37), or liver dysfunction (9.3%, *n*=33); or they used key medications such as antibiotics (61.6%, *n*=218) and nutritional support therapy (20.6%, *n*=73), or medications required TDM (20.3%, *n*=72), etc.

In this period, 427 DRPs were identified, and 486 pharmacy interventions were proposed by pharmacists to solve the problems during priority monitoring; 245 DRPs were identified, and 273 interventions related to drug therapy changes were proposed during consultation. For additional pharmacy services, the team made 93 individualized drug regimen recommendations for TDM patients or based on genetic test results, reported 21 cases of ADR, and provided 14 teaching sessions to the physicians and/or nurses in the SICU.

We compared the pharmacy-related services completed during 2017 (before PC model implementation) and the study period to assess if its implementation could improve the efficacy of PC team in providing patient care services. Under the guidance of the SICU PC model, the number of patients on closely monitoring by the PC team increased from 7.7 to 16.9 per month. The monthly pharmacy interventions made increased from 13.6 to 20.1 cases, while the consultations provided slightly decreased from 13.3 to 12.6 cases (see **Supplementary Figures 2** and **3**).

### Pharmacy Intervention

During the close monitoring of 354 SICU patients, 423 pharmacy intervention records were collected, and pharmacy interventions were suggested for 213 patients (60.2%). A total number of 427 DRPs were identified from 412 records (11 records related to non-medical problems were removed, see **Figure 1**), and pharmacists proposed 486 interventions to solve the DRPs with an acceptance rate of 97.3%. On average, interventions were made to 12.1 cases per 100 patient cases admitted to the unit; pharmacy interventions were proposed for 34.9% patients who are on priority monitoring, and 0.9 recommendations were made for each patient on the unit per month.

#### ME Classification

ME classification using NCC-MERP criteria was performed for the 427 DRPs identified during pharmacy intervention. Most of the DRPs were classified as category C (82.2%, *n*=351), followed by category D (7.5%, *n*=32) and E (5.6%, *n*=24), with the latter two causing potential harms or harms to patients. MEs in categories A and B only counted for 4.7% of the total (*n*=5, 15 respectively).

#### DRPs and Pharmacy Interventions During Close Patient Monitoring

Data analysis results of DRPs and causes related to all medications and antibiotics were shown in **Table 2**. Among 427 DRPs identified from all medications, the primary problems were treatment safety and effectiveness (69% in total), including “P2.1 Adverse drug event (possibly) occurring,” “P1.2 Effect of drug treatment not optimal,” and “P3.2 Unnecessary drug-treatment.” Medication classification results indicated that the top 3 medicines leading to DRPs were antibiotics (59.7%), parenteral nutrition (5.8%) and proton pump inhibitors (PPI, 2.7%), see ***Supplementary Materials*** (**Table 2** and **Figure 4**) for details.

**Table 2:** Number of drug-related problems and causes of all medicines and antibiotics during pharmacy intervention.

Description	All medicines		Antibiotics	
Problem	N	Proportion, %	N	Proportion, %
**P1 Treatment effectiveness**	146	34.2	107	42.1
P1.1 No effect of drug treatment	14	3.3	12	4.7
P1.2 Effect of drug treatment not optimal	109	25.5	87	34.3
P1.3 Untreated symptoms or indication	23	5.4	8	3.1
**P2 Treatment safety**	133	31.1	57	22.4
P2.1 Adverse drug event (possibly) occurring	133	31.1	57	22.4
**P3 Other**	148	34.7	90	35.4
P3.1 Problem with cost-effectiveness of the treatment	3	0.7	0	0.0
P3.2 Unnecessary drug-treatment	89	20.8	46	18.1
P3.3 Needs additional TDM	49	11.5	38	15.0
P3.4 Antibiotics De-escalation	7	1.6	6	2.4
**Total**	**427**	**100.0**	**254**	**100.0**
**Cause**	**N**	**Proportion, %**	**N**	**Proportion, %**
**C1 Drug selection**	198	41.3	96	33.6
C1.1 Inappropriate drug according to guidelines/formulary	45	9.4	36	12.6
C1.2 Inappropriate drug (within guidelines but otherwise contraindicated)	42	8.8	14	4.9
C1.3 No indication for drug	25	5.2	9	3.1
C1.4 Inappropriate combination of drugs, or drugs and herbal medications, or drugs and dietary supplements	13	2.7	1	0.3
C1.5 Inappropriate duplication of therapeutic group or active ingredient	12	2.5	8	2.8
C1.6 No or incomplete drug treatment in spite of existing indication	42	8.8	16	5.6
C1.7 Too many drugs prescribed for indication	5	1.0	3	1.0
C1.8 Necessary genetic testing before drug initiation	14	2.9	9	3.1
**C2 Drug form**	7	1.5	0	0
C2.1 Inappropriate drug form (for this patient)	7	1.5	0	0
**C3 Dose selection**	139	29.0	106	37.1
C3.1 Drug dose too low	40	8.4	33	11.5
C3.2 Drug dose too high	64	13.4	42	14.7
C3.3 Dosage regimen not frequent enough	33	6.9	30	10.5
C3.4 Dosage regimen too frequent	2	0.4	1	0.3
**C4 Treatment duration**	65	13.6	33	11.5
C4.2 Duration of treatment too long	65	13.6	33	11.5
**C6 Drug use process**	4	0.8	1	0.3
C6.1 Inappropriate timing of administration or dosing intervals	4	0.8	1	0.3
**C8 Patient transfer related**	2	0.4	0	0
C8.3 Discharge/transfer information about medication incomplete or missing	2	0.4	0	0
**C9 Other**	64	13.4	50	17.5
C9.1 No or inappropriate outcome monitoring (incl. TDM)	64	13.4	50	17.5
**Total**	**479**	**100.0**	**286**	**100.0**

TDM, therapeutic drug monitoring.

During the 21-month study period, a total number of 486 interventions were proposed by pharmacists to solve the DRPs. About four fifths (81.7%) of the interventions were made at drug level, mainly including “I3.5 Drug paused or stopped” (27.4%), “I3.2 Dosage changed” (26.5%) and “I3.1 Drug changed” (15.8%), see **Supplementary Figure 5**. Less than one fifth (17.9%) interventions (such as ordering labs or genetic tests) were made at prescriber level, of which 98% were proposed to the prescriber. Only 0.4% interventions were proposed at patient level as most of the SICU patients were sedated and not able to communicate.

#### Causes of Identified DRPs

The analysis of 479 DRP causes of all medications showed “C1 Drug selection” caused the highest proportion of DRPs (41.3%), followed by “C3 Dose selection” and”C4 Treatment duration.” The major sub-category of DRP causes were “C4.2 Duration of treatment too long,” “C3.2 Drug dose too high,” and “C9.1 No or inappropriate outcome monitoring (incl. TDM)” (see **Supplementary Figure 6**).

#### Relationship Between DRPs and Causes

**Figure 2:**
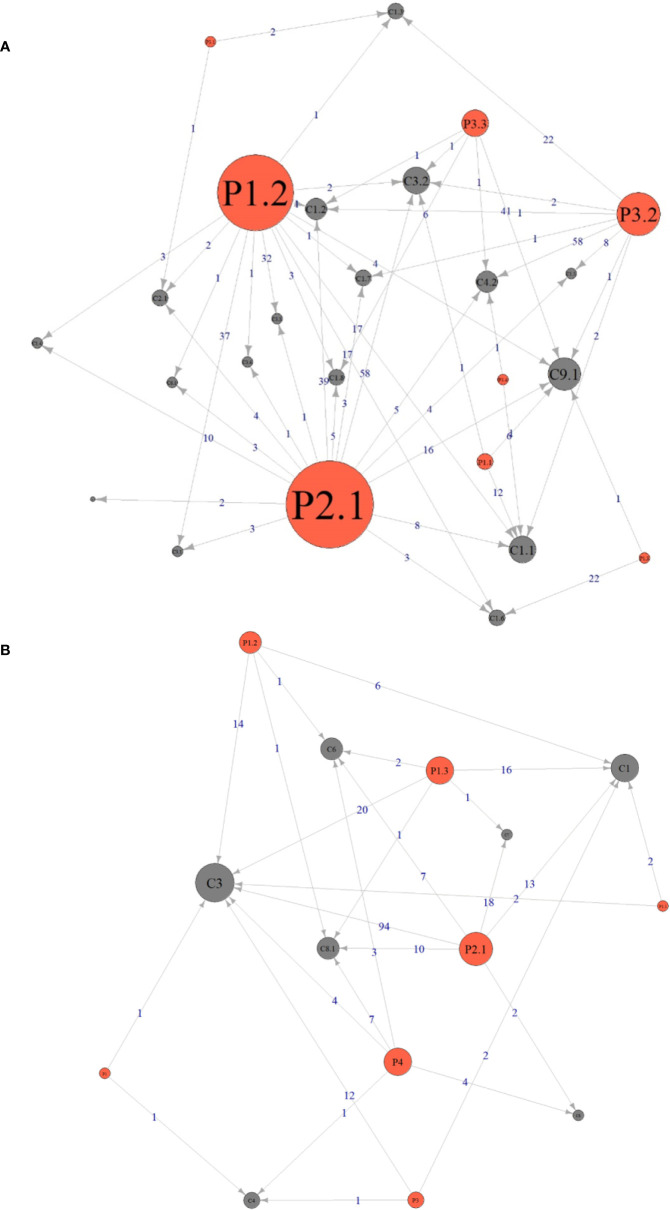
Relationship between the drug-related problems and causes identified during pharmacy intervention **(A)** and consultation **(B)**. The size of the circle indicates how many times this DRPs or Cause was identified. An arrow pointing from P (Problems) to C (Causes) means the problem was caused by the corresponding cause and the number on the line indicates the frequency of this causal relationship. The definitions of Px.x (or Px) and Cx.x (or Cx) during pharmacy intervention and consultation were listed in **Tables 2** and **3**, respectively.

The relationship between the DRPs and causes was analyzed and shown in **Figure 2**. During pharmacy intervention, the main cause leading to adverse drug events (P2.1) was drug dose too high (C3.2, *n*=58), contraindicated drug regimen (C1.2, *n*=39), and no or inappropriate outcome monitoring (C9.1, *n*=16). The main Causes of not optimal drug treatment (P1.2) was drug dose too low (C3.1, *n*=37) and dosage regimen not frequent enough (C3.3, *n*=32). Unnecessary drug treatment (P3.2) was mainly caused by drug duration too long (C4.2, *n*=58). The relationship between the DRPs and causes in antibiotic use was shown in **Supplementary Figure 7**.

### Pharmacy Consultation

During the 21-month study period, 265 pharmacy consultation records were collected, and recommendations related to drug therapy changes were suggested for 131 patients, including nine children and five perinatal women. The mean age of the patient population was 61 (IQR, 42, 74) years old, and 58.1% of patients were males (*n* = 105). Notably, 29% of the patients had renal dysfunction. Only 243 records were included for DRP classification (22 records on non-clinical issues or from non-SICU departments removed, see **Supplementary Figure 1**), from which 245 DRPs were identified. A total number of 273 interventions related to drug therapy changes were proposed by pharmacists (consultations provided drug information only were not counted) with an acceptance rate of 99.3%. On average, pharmacists completed 12.6 consultations and proposed 13 interventions related to drug therapy changes per month.

#### DRPs and Causes Identified During Pharmacy Consultation

Data analysis results of DRPs and causes related to all medications and antibiotics were shown in **Table 3**. Among 245 DRPs identified during pharmacy consultation, the proportion of “P2 Treatment safety” (58.4%) was significantly higher than others. The major sub-category of DRPs were “P2.1 Adverse drug event (possibly) occurring,” “P1.3 Untreated symptoms or indication,” and “P1.2 Effect of drug treatment not optimal” (see **Supplementary Figures 8** and **9**). The medication classification results of all DRPs indicated the top 3 medicines being consulted were antibiotics (62.2%, *n*=153), antifungal drugs (8.53%, *n*=21), and antiepileptic drugs (3.66%, *n*=9), see **Supplementary Table 3** for details.

**Table 3:** Number of drug-related problems and causes of all medicines and antibiotic medicines of pharmacy consultation.

	All medicines		Antibiotics	
Problem	N	Proportion, %	N	Proportion, %
**P1 Treatment effectiveness**	68	27.8	39	25.5
P1.1 No effect of drug treatment	4	1.6	3	2.0
P1.2 Effect of drug treatment not optimal	22	9.0	16	10.5
P1.3 Untreated symptoms or indication	40	16.3	19	12.4
P1.4 Other	2	0.8	1	0.7
**P2 Treatment safety**	143	58.4	99	64.7
P2.1 Adverse drug event (possibly) occurring	143	58.4	99	64.7
**P3 Treatment safety and cost-effectiveness**	15	6.1	12	7.8
**P4 Other**	19	7.8	3	2.0
**Total**	**245**	**100.0**	**153**	**100.0**
**Cause**	**N**	**Proportion, %**	**N**	**Proportion, %**
**C1 Drug selection**	39	15.9	21	13.7
**C3 Dose selection**	147	59.8	114	74.5
**C4 Treatment duration**	3	1.2	2	1.3
**C6 Drug use process**	13	5.3	4	2.6
**C7 Drug-related side effects/Drug-induced diseases**	19	7.7	9	5.9
**C8 Other**	25	10.2	3	2.0
**Total**	**246**	**100.0**	**153**	**100.0**

The analysis result of 246 DRP Causes showed “C3 Dose selection” caused the highest proportion of DRPs (59.8%), followed by “C1 Drug selection” and “C7 Drug-related side effects/Drug-induced diseases.”

#### Relationship Between DRPs and Causes

The relationship between DRPs and causes was analyzed and shown in **Figure 2**. During pharmacy consultation, the main Cause leading to P2.1 Adverse drug event was “C3 Dose selection” (*n*=94), followed by “C7 Drug-related diseases” (*n*=18) and “C1 Drug selection” (*n*=13), and the main Cause leading to “P1.3 Untreated symptoms or indications” was “C3 Dose selection” (*n*=20) and “C1 Drug selection” (*n*=16). For the relationship between DRPs and causes in antibiotic use, see **Supplementary Figure 10**.

### Comparison of (Possible) DRPs Identified During Pharmacy Interventions and Consultations

**Figure 3:**
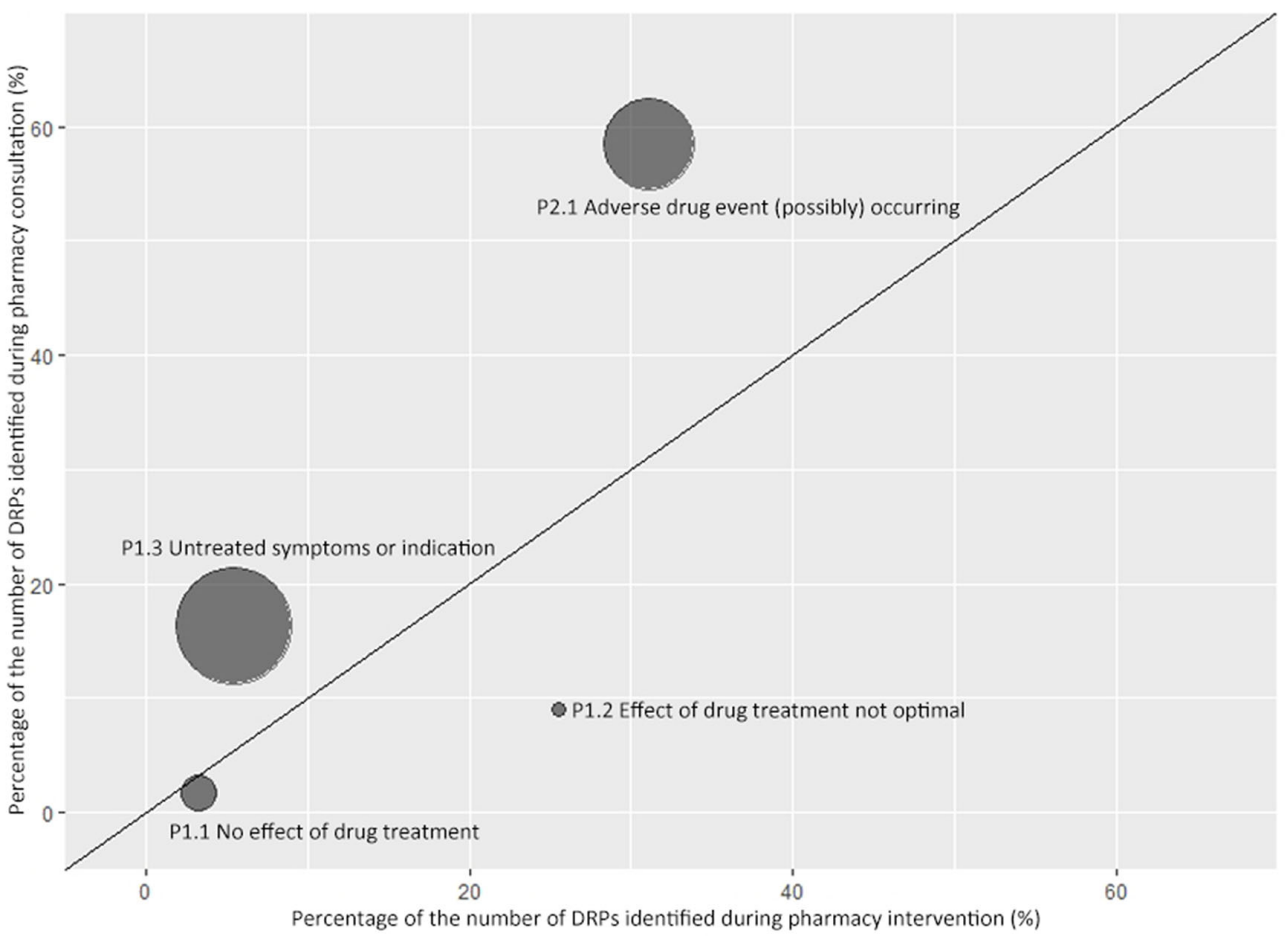
Comparison of drug-related problems identified during pharmacy consultation and intervention. A symmetrical bubble chart was used to describe the ratio of the proportion of DRPs identified during pharmacy consultation and intervention. The horizontal axis indicates the proportion of the corresponding Problem in all DRPs identified during pharmacy intervention. The vertical axis indicates the proportion of the corresponding Problem in all DRPs identified during pharmacy consultation. The size of the bubble is proportional to the ratio between y and x axes. The oblique line shown in the figure is a straight line with a slope of 1.

In this session, four sub-categories of DRPs were compared as they had the same definition in pharmacy intervention and consultation classification. As shown in **Figure 3**, bubbles above the line indicate that problem appears more frequently during medication consultation than pharmacy intervention. The order of ratio from high to low was 3.02 (P1.3 Untreated symptoms or indication; 16.3%/5.4%), 1.88 (P2.1 Adverse drug event (possibly) occurring; 58.4%/31.1%), 0.48 (P1.1 No effect of drug treatment; 1.6%/3.3%), and 0.35 (P1.2 Effect of drug treatment not optimal; 9%/25.5%).

Two bubbles representing untreated symptoms or indications and adverse drug events, respectively, are above the oblique line, which indicates physicians tended to ask pharmacists for help when they had questions in these two areas. The other two bubbles below the line representing drug treatment showing no effect or effect not optimized, which indicates pharmacists tended to solve more DRPs in these two areas than they were asked by physicians. “P3.1 Problem with cost-effectiveness of the treatment” and “P3.2 Unnecessary drug-treatment” were incomparable as they only occurred during pharmacy intervention.

## Discussion

This is a pilot study for evaluating a newly developed PC model and identifying DRPs in ICU under a funding support. In fact, it was driven by the actual clinical need of effective and standardized pharmacy services in the ICU setting, and the PC model is now being used in the daily practice of the SICU pharmacists in our hospital. In this prospective study, we showed a dedicated pharmacy team in the SICU could work more efficiently in patients’ monitoring and identifying DRPs and preventable MEs through implementing a PC model. The number of pharmacy interventions proposed on medication orders increased from 13.6 cases monthly to 20.1 cases monthly, and the patients on priority monitoring by the PC team doubled.

The primary results of our study are in line with previous reports. (1) The incidence rates of interventions is 12.1 cases per 100 patient admissions, being similar to the rate of preventable ordering adverse drug events as 14.7 per 100 patients in previous report (Leape et al., 1999). (2) ADE occurring (31.1%) was the most frequently detected DRPs and more than four fifths of DRPs were due to drug selection, dose selection, and treatment duration, being in line with an evaluation of pharmacists’ interventions in a Swiss study (Reinau et al., 2019). (3) Drugs that most frequently caused DRPs during intervention were antibiotics, showing a similar composition but with a higher percentage than that of previous studies (Klopotowska et al., 2010; Johansen et al., 2016; Reinau et al., 2019). (4) More than 97% of advice given by the pharmacist were accepted or taken into consideration, being much higher than previously reported in a mixed Norwegian ICU (87%) (Johansen et al., 2016), but the same as it in a Swiss university hospital (97.8%) (Reinau et al., 2019). Though the priority monitoring was only performed for one-fifths of SICU patients due to limited human resources, the primary results of this study were consistent with other studies covering the total patients. It suggests that the PC model implemented in this study is efficient in identifying high-risk patient population that pharmacists should focus on; and the establishment of such practice models can be beneficial to institutions under limited human resources in identifying high-risk patients and the majority of DRPs.

The PC model was set up on the basis of pharmacy services and requirements in a position paper (Rudis and Brandl, 2000). More importantly, it neatly blended previous experience and hospital characters into local practices. Having the strongest medical teams in surgical areas such as general spine, obstetrics and gynecology, plastic surgery, and general surgery among China, our hospital carries out about 76,000 surgical cases per year with some of them being very difficult and creative (Xu et al., 2016). Thus, SICU is an important platform for perioperative support of high-risk patients. Compared with patients in medicine ICU, SICU patients apply anticoagulants and parenteral nutrition more frequently, and are more likely to develop multiple organ dysfunction syndrome owing to sepsis, trauma, post cardiopulmonary resuscitation, and obstetrics complications, etc. Thus, this PC model, aiming at guiding pharmacists’ daily practice under limited human resources, was designed to cover those populations and their medication therapies. In addition to commonly used medications in SICU, we added certain medications on the key monitoring list of the hospital to ensure their appropriate use, such as traditional Chinese injections. The findings of the study suggested it was an effective PC model in identifying DRPs and improving the efficacy of pharmacy services, which provided lessons for the process of setting up a PC model that concreting guidelines with adjustments and details according to local characteristics.

There are two important findings in this study, and the first one is related to antibiotic use. Similar to previous reports, we found antibiotics was the top medicine-related DRPs during both pharmacy intervention (59.7%) and consultation (62.2%). This can be primarily explained by the high prevalence of infectious diseases in ICU setting (Klopotowska et al., 2010; Johansen et al., 2016; Reinau et al., 2019). Moreover, our study showed a much higher proportion of DRPs caused by antibiotics (comparing with 48.9% in a Swiss ICU (Reinau et al., 2019) and 22% in a mixed Norwegian ICU (Johansen et al., 2016)), which can be explained partly by the current antibiotic management strategies in China. A series of measures were adopted by Chinese government to improve antibiotic use over the past decades (State Council of China, 2009; Van Boeckel et al., 2014), including the establishment of national guidelines (Ministry of Health, 2004; Ministry of Health, 2015), the surveillance networks for antibiotic use and antimicrobial resistance (Ministry of Health, 2005), and a 3-year national level regulatory campaign launched in 2011 (Ma et al., 2016). Following these regulations, an antibiotic administrative group was set up in the studied hospital for antibiotic use monitoring, and they announced the drug utilization and re-evaluation results of antibiotics prescription monthly. As a result, both physicians and pharmacists tended to pay more attention on antibiotic use to avoid or identify DRPs promptly and solved them internally in order to meet hospital regulations and governmental policies.

Additionally, among the total 286 DRPs related to antibiotics during intervention, the most frequent causes were dose selection, inappropriate outcome monitoring and inappropriate drug according to guidelines, indicating the potential areas that pharmacists could contribute in. In practice, clinical pharmacists can fill the gap by working as an integral member in the MDT and offering optimal antimicrobial therapies according to infection sites, pathogens, pharmacokinetics/pharmacodynamics (PK/PD) parameters of antibiotics, and patients’ renal or liver function (Zhou et al., 2016; Wang et al., 2019). In addition to consulting pharmacists on antibiotic selection and dosing, it may also be important to set up more targeted courses to equip ICU physicians with essential knowledge on antibiotic use (Guan et al., 2019; Wushouer et al., 2020).

The second important findings was the difference of identified DRPs between pharmacy interventions and consultations. Through comparison, we found that SICU physicians tended to seek recommendations actively from clinical pharmacists in face of identifying ADRs, choosing a cost-effectiveness treatment, and requiring a dosage regimen when a new therapy initiates. However, under circumstances as selecting a drug for untreated symptoms or indications, optimizing therapy or discontinuing unnecessary treatment, SICU physicians tended to accept recommendations passively from clinical pharmacists.

There were three possible reasons for this. First, with a better understanding of diseases and as a result of experimental teaching, SICU physicians relied more on their accumulated clinical experience instead of updated guidelines that clinical pharmacists attach importance to when evaluating treatment effectiveness. Second, owing to the legal considerations of off label-use (Lenk and Duttge, 2014), and the uncertainty on the applicability and safety of dosage recommendations of international guidelines on local populations (Koga et al., 2012; Kim et al., 2015), SICU physicians were prone to a therapeutic option with approved indications and dosage regimens, even if there was a potential better option. Thirdly, in the selected SICU where most patients were in perioperative period, it is common for the operators to participate in making clinical decisions as consultants or co-attending. ICU physicians might feel pressure when they held different opinions on treatment plans, and unnecessary medication orders could be carried out in this way. These situations bring great opportunities for pharmacists to get involved, communicate with both sides and ensure medication safety (Rudis and Brandl, 2000; Kane et al., 2003; Penm et al., 2014a; Tasaka et al., 2018; Reinau et al., 2019).

At present, pharmacists have been recognized by physicians as an essential member of the MDT, but fully understanding of each other’s skills and needs is still needed. SICU physicians should learn more about pharmacist capabilities in drug selection and regimen design, and pharmacists should continue improving their knowledge on therapeutics and medication safety to support physicians better. Finally, ADE occurring were the most frequent DRPs both in pharmacy intervention and consultation. It suggests that both physicians and pharmacists should continue strengthening a close cooperation in drug safety.

The study has three strengths. To start with, it is the first study to evaluate an ICU PC model in China. We carried out a prospective study and enrolled 354 consecutive SICU patients under the model developed by an experienced pharmacist team. According to the results, it may be generally applied to other ICU departments, especially those under limited human resources. Secondly, three researchers completed the DRP classification to guarantee the accuracy. Two of them took responsibility for the categorization of DRP types and subtypes, and a pilot test was performed to ensure they have the same understanding of the classification system. Thirdly, our study creatively compared the DRPs identified during pharmacy interventions and consultations, and showed the gap between pharmacy services currently being provided and the needs of physicians. The result will not only help physicians better understand the scope of pharmacy service beyond drug safety, but also guide pharmacists during their daily practice by reassuring the clinical needs of physicians.

The study also has two limitations. Firstly, data collection was mostly performed by the leading pharmacist, which may lead to information bias and the possibility of underestimating DRPs incidence. Nevertheless, three researchers participated in the DRP classification to minimize the bias of the study results. Secondly, the original DRP system cannot cover every PC point; we therefore developed a modified DRP system with slight changes to capture the key DRP types and causes.

## Conclusions

This study revealed the most common DRPs in the SICU setting of a Chinese comprehensive tertiary hospital during pharmacy interventions and consultations. Our results indicate that the establishment and implementation of an ICU PC mode is beneficial for guiding pharmacy practice and improving efficacy especially when human resource is limited. Additionally, physicians and pharmacists should continue their efforts in ensuring drug safety and get a better understanding of the scope of PC practice and clinical need in order to achieve a deeper cooperation in MDTs and improve the quality of ICU patient care together in the long run.

## Author’s Note

The authors confirm that the Principal Investigator for this paper is X-XL and that she had direct clinical responsibility for patients.

## Data Availability Statement

The raw data supporting the conclusions of this article will be made available by the authors. Please send a request to the principle investigator of this article. The steering committee of this study will discuss all requests and decide on the basis of the scientific rigor of proposal whether data sharing is appropriate. All applicants are asked to sign a data access agreement.

## Ethics Statement

This study was approved by the Ethical Review Board of Peking University Health Science Center (IRB00001052-20014). Written informed consent for participation was not required for this study in accordance with the national legislation and the institutional requirements.

## Author Contributions

All authors contributed to the article and approved the submitted version. X-XL: Conceptualization, methodology, investigation, writing—original draft. S-QZ: Conceptualization, methodology, data curation, writing—original draft. J-HG: Data curation, ethics application. TH: Formal analysis, visualization. FL: Supervision, Writing—review and editing. Q-GG: Supervision. BL: Investigation. CL: Investigation. MY: Supervision. Y-FQ: Data curation. R-SZ: Validation, funding acquisition. L-WS: Validation, funding acquisition.

## Funding 

This work was supported by the National Science and Technology Major Project of China (grant number 2018ZX09721003-001-002, 2018 and 2017ZX09304012, 2017).

## Conflict of Interest

The authors declare that the research was conducted in the absence of any commercial or financial relationships that could be construed as a potential conflict of interest.

## References

[B1] Anderson R. D. (1992). Mirror to ASHP: 1942-1992. Am. J. Hosp. Pharm. 49 (8), 1925–1935. 10.1093/ajhp/49.8.1925 1442835

[B2] Borthwick M. Barton G. Bourne R. S. McKenzie C. (2018). Critical care pharmacy workforce: UK deployment and characteristics in 2015. Int. J. Pharm. Pract. 26 (4), 325–333. 10.1111/ijpp.12408 29024199

[B3] Brennan T. A. Leape L. L. Laird N. M. Hebert L. Localio A. R. Lawthers A. G. (1991). Incidence of adverse events and negligence in hospitalized patients. Results of the Harvard Medical Practice Study I. N. Engl. J. Med. 324 (6), 370–376. 10.1056/NEJM199102073240604 1987460

[B4] Brixey J. Johnson T. R. Zhang J. (2002). Evaluating a medical error taxonomy. Proc. AMIA Symp. 71–75. 12463789 PMC2244554

[B5] Chou R. Gordon D. B. de Leon-Casasola O. A. Rosenberg J. M. Bickler S. Brennan T. (2016). Management of Postoperative Pain: A Clinical Practice Guideline From the American Pain Society, the American Society of Regional Anesthesia and Pain Medicine, and the American Society of Anesthesiologists’ Committee on Regional Anesthesia, Executive Committee, and Administrative Council. J. Pain 17 (2), 131–157. 10.1016/j.jpain.2015.12.008 26827847

[B6] Frontera J. A. Lewin J. J. 3rd. Rabinstein A. A. Aisiku I. P. Alexandrov A. W. Cook A. M. (2016). Guideline for Reversal of Antithrombotics in Intracranial Hemorrhage: Executive Summary. A Statement for Healthcare Professionals From the Neurocritical Care Society and the Society of Critical Care Medicine. Crit. Care Med. 44 (12), 2251–2257. 10.1097/CCM.0000000000002057 27858808

[B7] Garrouste-Orgeas M. Timsit J. F. Vesin A. Schwebel C. Arnodo P. Lefrant J. Y. (2010). Selected medical errors in the intensive care unit: results of the IATROREF study: parts I and II. Am. J. Respir. Crit. Care Med. 181 (2), 134–142. 10.1164/rccm.200812-1820OC 19875690

[B8] Garrouste-Orgeas M. Perrin M. Soufir L. Vesin A. Blot F. Maxime V. (2015). The Iatroref study: medical errors are associated with symptoms of depression in ICU staff but not burnout or safety culture. Intensive Care Med. 41 (2), 273–284. 10.1007/s00134-014-3601-4 25576157

[B9] Garrouste-Orgeas M. Flaatten H. Moreno R. (2016). Understanding medical errors and adverse events in ICU patients. Intensive Care Med. 42 (1), 107–109. 10.1007/s00134-015-3968-x 26248952

[B10] Griese-Mammen N. Hersberger K. E. Messerli M. Leikola S. Horvat N. van Mil J. W. F. (2018). PCNE definition of medication review: reaching agreement. Int. J. Clin. Pharm. 40 (5), 1199–1208. 10.1007/s11096-018-0696-7 30073611

[B11] Guan X. Tian Y. Song J. Zhu D. Shi L. (2019). Effect of physicians’ knowledge on antibiotics rational use in China’s county hospitals. Soc. Sci. Med. 224, 149–155. 10.1016/j.socscimed.2019.01.049 30784853

[B12] Hu M. Yee G. Zhou N. Yang N. Jiang X. Klepser D. (2014). Development and current status of clinical pharmacy education in China. Am. J. Pharm. Educ. 78 (8), 157. 10.5688/ajpe788157 25386022 PMC4226294

[B13] Johansen E. T. Haustreis S. M. Mowinckel A. S. Ytrebo L. M. (2016). Effects of implementing a clinical pharmacist service in a mixed Norwegian ICU. Eur. J. Hosp. Pharm. 23 (4), 197–202. 10.1136/ejhpharm-2015-000751 31156848 PMC6451479

[B14] Kane S. L. Weber R. J. Dasta J. F. (2003). The impact of critical care pharmacists on enhancing patient outcomes. Intensive Care Med. 29 (5), 691–698. 10.1007/s00134-003-1705-3 12665997

[B15] Kari H. Kortejarvi H. Airaksinen M. Laaksonen R. (2018). Patient involvement is essential in identifying drug-related problems. Br. J. Clin. Pharmacol. 84 (9), 2048–2058. 10.1111/bcp.13640 29774588 PMC6089828

[B16] Kellum J. A. Lameire N. Group K. A. G. W. (2013). Diagnosis, evaluation, and management of acute kidney injury: a KDIGO summary (Part 1). Crit. Care 17 (1), 204. 10.1186/cc11454 23394211 PMC4057151

[B17] Kim N. H. So M. S. Kang J. G. Cho D. S. Byrne C. D. Lee S. J. (2015). Application of new guidelines for the primary prevention of atherosclerotic cardiovascular disease in a Korean population. J. Atheroscler. Thromb. 22 (3), 293–303. 10.5551/jat.26682 25284329

[B18] Klopotowska J. E. Kuiper R. van Kan H. J. de Pont A. C. Dijkgraaf M. G. Lie A. H. L. (2010). On-ward participation of a hospital pharmacist in a Dutch intensive care unit reduces prescribing errors and related patient harm: an intervention study. Crit. Care 14 (5), R174. 10.1186/cc9278 20920322 PMC3219276

[B19] Koga M. Shiokawa Y. Nakagawara J. Furui E. Kimura K. Yamagami H. (2012). Low-dose intravenous recombinant tissue-type plasminogen activator therapy for patients with stroke outside European indications: Stroke Acute Management with Urgent Risk-factor Assessment and Improvement (SAMURAI) rtPA Registry. Stroke 43 (1), 253–255. 10.1161/STROKEAHA.111.631176 21960585

[B20] Leape L. L. Brennan T. A. Laird N. Lawthers A. G. Localio A. R. Barnes B. A. (1991). The nature of adverse events in hospitalized patients. Results of the Harvard Medical Practice Study II. N. Engl. J. Med. 324 (6), 377–384. 10.1056/NEJM199102073240605 1824793

[B21] Leape L. L. Cullen D. J. Clapp M. D. Burdick E. Demonaco H. J. Erickson J. I. (1999). Pharmacist participation on physician rounds and adverse drug events in the intensive care unit. JAMA 282 (3), 267–270. 10.1001/jama.282.3.267 10422996

[B22] Lee H. Ryu K. Sohn Y. Kim J. Suh G. Y. Kim E. (2019). Impact on Patient Outcomes of Pharmacist Participation in Multidisciplinary Critical Care Teams: A Systematic Review and Meta-Analysis. Crit. Care Med. 47 (9), 1243–1250. 10.1097/CCM.0000000000003830 31135496

[B23] Leendertse A. J. Egberts A. C. Stoker L. J. van den Bemt P. M. Group H. S. (2008). Frequency of and risk factors for preventable medication-related hospital admissions in the Netherlands. Arch. Intern. Med. 168 (17), 1890–1896. 10.1001/archinternmed.2008.3 18809816

[B24] Lenk C. Duttge G. (2014). Ethical and legal framework and regulation for off-label use: European perspective. Ther. Clin. Risk Manag. 10, 537–546. 10.2147/TCRM.S40232 25050064 PMC4103928

[B25] Li X. X. Zhang C. Liu F. Zhai S. D. Yang Y. H. (2019). Application of PDCA cycle method in the establishment and implementation of quality control system in clinical pharmacy. Chin. J. Hosp. Pharm. 39 (22), 2347–2350. 10.13286/j.cnki.chinhosppharmacyj.2019.22.19

[B26] Liu F. Zhang T. Zhang X. L. Zhu Z. Zhai S. D. (2018). Development of High-Alert Medication List Based on Expert Consensus and Healthcare Workers Investigation. Chin. Pharm. J. 53 (17), 1523–1528. 10.11669/cpj.2018.17.018

[B27] Ma X. Xie J. Yang Y. Guo F. Gao Z. Shao H. (2016). Antimicrobial stewardship of Chinese ministry of health reduces multidrug-resistant organism isolates in critically ill patients: a pre-post study from a single center. BMC Infect. Dis. 16 (1), 704. 10.1186/s12879-016-2051-8 27887595 PMC5123232

[B28] McClave S. A. Taylor B. E. Martindale R. G. Warren M. M. Johnson D. R. Braunschweig C. (2016). Guidelines for the Provision and Assessment of Nutrition Support Therapy in the Adult Critically Ill Patient: Society of Critical Care Medicine (SCCM) and American Society for Parenteral and Enteral Nutrition (A.S.P.E.N.). JPEN J. Parenter. Enteral. Nutr. 40 (2), 159–211. 10.1177/0148607115621863 26773077

[B29] Ministry of Health (2004). Notification on issuing National Guiding Principles for Antimicrobial Clinical Use. Available at: http://www.nhc.gov.cn/wjw/gfxwj/201304/2c850f3dc54244ca846d8a17baf3613d.shtml (Accessed 17 May 2020).

[B30] tMinistry of Health (2005). Notification on Establishing China Antimicrobial Resistance Surveillance System and Center for Antibacterial Surveillance. Available at: http://www.nhc.gov.cn/zwgk/jdjd/201304/b0da0ebfc7b3428f98d435a29cfa4250.shtml (Accessed 17 May 2020).

[B31] Ministry of Health (2015). Notification on issuing National Guiding Principles (2015) for Antimicrobial Clinical Use. Available at: http://www.nhc.gov.cn/yzygj/s3593/201508/c18e1014de6c45ed9f6f9d592b43db42.shtml (Accessed 17 May 2020).

[B32] Penm J. Li Y. Zhai S. Hu Y. Chaar B. Moles R. (2014a). The impact of clinical pharmacy services in China on the quality use of medicines: a systematic review in context of China’s current healthcare reform. Health Policy Plan 29 (7), 849–872. 10.1093/heapol/czt067 24056897

[B33] Penm J. Moles R. Wang H. Li Y. Chaar B. (2014b). Factors affecting the implementation of clinical pharmacy services in China. Qual. Health Res. 24 (3), 345–356. 10.1177/1049732314523680 24562375

[B34] Qu C. Meng L. Wang N. Chen Y. Yang X. Wang J. (2019). Identify and categorize drug-related problems in hospitalized surgical patients in China. Int. J. Clin. Pharm. 41 (1), 13–17. 10.1007/s11096-018-0777-7 30610549

[B35] Reinau D. Furrer C. Stampfli D. Bornand D. Meier C. R. (2019). Evaluation of drug-related problems and subsequent clinical pharmacists’ interventions at a Swiss university hospital. J. Clin. Pharm. Ther. 44 (6), 924–931. 10.1111/jcpt.13017 31408206

[B36] Rhodes A. Evans L. E. Alhazzani W. Levy M. M. Antonelli M. Ferrer R. (2017). Surviving Sepsis Campaign: International Guidelines for Management of Sepsis and Septic Shock: 2016. Crit. Care Med. 45 (3), 486–552. 10.1097/CCM.0000000000002255 28098591

[B37] Rudis M. I. Brandl K. M. (2000). Position paper on critical care pharmacy services. Society of Critical Care Medicine and American College of Clinical Pharmacy Task Force on Critical Care Pharmacy Services. Crit. Care Med. 28 (11), 3746–3750. 10.1097/00003246-200011000-00037 11098984

[B38] Schneider P. J. Pedersen C. A. Ganio M. C. Scheckelhoff D. J. (2019). ASHP national survey of pharmacy practice in hospital settings: Workforce-2018. Am. J. Health Syst. Pharm. 76 (15), 1127–1141. 10.1093/ajhp/zxz102 31361871

[B39] State Council of China (2009). Opinions of the CPC Central Committee and the State Council on Deepening the Health Care System Reform. Available at: http://www.china.org.cn/government/scio-press-conferences/2009-04/09/content_17575378.htm (Accessed 17 May 2020).

[B40] Tasaka Y. Tanaka A. Yasunaga D. Asakawa T. Araki H. Tanaka M. (2018). Potential drug-related problems detected by routine pharmaceutical interventions: safety and economic contributions made by hospital pharmacists in Japan. J. Pharm. Health Care Sci. 4, 33. 10.1186/s40780-018-0125-z 30564432 PMC6293536

[B41] Taskforce D. A. S. Baron R. Binder A. Biniek R. Braune S. Buerkle H. (2015). Evidence and consensus based guideline for the management of delirium, analgesia, and sedation in intensive care medicine. Revision 2015 (DAS-Guideline 2015) - short version. Ger. Med. Sci. 13, Doc19. 10.3205/000223 26609286 PMC4645746

[B42] Van Boeckel T. P. Gandra S. Ashok A. Caudron Q. Grenfell B. T. Levin S. A. (2014). Global antibiotic consumption 2000 to 2010: an analysis of national pharmaceutical sales data. Lancet Infect. Dis. 14 (8), 742–750. 10.1016/S1473-3099(14)70780-7 25022435

[B43] Walsh E. K. Hansen C. R. Sahm L. J. Kearney P. M. Doherty E. Bradley C. P. (2017). Economic impact of medication error: a systematic review. Pharmacoepidemiol. Drug Saf. 26 (5), 481–497. 10.1002/pds.4188 28295821

[B44] Wang H. Wang H. Yu X. Zhou H. Li B. Chen G. (2019). Impact of antimicrobial stewardship managed by clinical pharmacists on antibiotic use and drug resistance in a Chinese hospital 2010-2016: a retrospective observational study. BMJ Open 9 (8), e026072. 10.1136/bmjopen-2018-026072 PMC6687004 31377693

[B45] Wei Y. H. Shi L. W. Shao H. Nie X. Y. Li X. X. Yang W. (2011). Current status and analysis of Chinese clinical pharmacists. Chin. J. New Drug 20 (9), 844–848. CNKISUNZXYZ.0.2011.09.019

[B46] Wushouer H. Wang Z. Tian Y. Zhou Y. Zhu D. Vuillermin D. (2020). The impact of physicians’ knowledge on outpatient antibiotic use: Evidence from China’s county hospitals. Med. (Baltimore) 99 (3), e18852. 10.1097/MD.0000000000018852 PMC7220442 32011504

[B47] Xu N. Wei F. Liu X. Jiang L. Cai H. Li Z. (2016). Reconstruction of the Upper Cervical Spine Using a Personalized 3D-Printed Vertebral Body in an Adolescent With Ewing Sarcoma. Spine (Phila Pa 1976) 41 (1), E50–E54. 10.1097/BRS.0000000000001179 26335676

[B48] Ye Z. K. Chen Y. L. Chen K. Zhang X. L. Du G. H. He B. (2016). Therapeutic drug monitoring of vancomycin: a guideline of the Division of Therapeutic Drug Monitoring, Chinese Pharmacological Society. J. Antimicrob. Chemother. 71 (11), 3020–3025. 10.1093/jac/dkw254 27494905

[B49] Zhou L. Ma J. Gao J. Chen S. Bao J. (2016). Optimizing Prophylactic Antibiotic Practice for Cardiothoracic Surgery by Pharmacists’ Effects. Medicine (Baltimore) 95 (9), e2753. 10.1097/MD.0000000000002753 26945362 PMC4782846

[B50] Zhou X. Qiu F. Wan D. Sun S. Yao G. Liu Y. (2019). Nutrition support for critically ill patients in China: role of the pharmacist. Asia Pac. J. Clin. Nutr. 28 (2), 246–251. 10.6133/apjcn.201906_28(2).0006 31192553

