# Supplementary material for: Drug-Related Problems Identified During Pharmacy Intervention and Consultation: Implementation of an Intensive Care Unit Pharmaceutical Care Model
Source: Front Pharmacol. 2020 Sep 11;11:571906. doi: 10.3389/fphar.2020.571906 (PMC7516263; doi:10.3389/fphar.2020.571906)
Supplement: Supplementary file 1 [file Presentation_1.pdf]

Supplementary Supplementary Table 1. Classification for drug related problems

Code	Domains	Code	Problem/ Cause/ Intervention		
			**Original version (V9.0)**	**Modified version for intervention**	**Modified version for consultation**
Problems (also potential)					
**P1**	**Treatment effectiveness**<br>There is a (potential) problem with the (lack of) effect of the pharmacotherapy	**P1.1**	No effect of drug treatment		
		**P1.2**	Effect of drug treatment not optimal		
		**P1.3**	Untreated symptoms or indication		
**P2**	**Treatment safety**<br>Patient suffers, or could suffer, from an adverse drug event	**P2.1**	Adverse drug event (possibly) occurring		
**P3** [#]	**Other**	**P3.1**	Problem with cost-effectiveness of the treatment		**Treatment safety and efficacy**
		**P3.2**	Unnecessary drug-treatment		
		**P3.3** [*]	**Unclear problem/complaint. Further clarification necessary**	**Needs Supplementary drug therapeutic monitoring (TDM)**	
		**P3.4** [*]	**/**	**Antibiotics De-escalation**	
**P4** [#]	**Other**	/	/	/	Other
Causes (including possible cases for potential problems)					
**C1** [#]	**Drug selection**<br>The cause of the DRP can be related to the selection of the drug	**C1.1**	Inappropriate drug according to guidelines/formulary		**Drug/Drug regimen selection**
		**C1.2**	Inappropriate drug (within guidelines but otherwise contra-indicated)		
		**C1.3**	No indication for drug		
		**C1.4**	Inappropriate combination of drugs, or drugs and herbal medications, or drugs and dietary supplements		
		**C1.5**	Inappropriate duplication of therapeutic group or active ingredient		
		**C1.6**	No or incomplete drug treatment in spite of existing indication		

Code	Domains	Code	Problem/ Cause/ Intervention		
			Original version (V9.0)	Modified version for intervention	Modified version for consultation
		C1.7	Too many drugs prescribed for indication		
		C1.8 *	/	Necessary genetic testing before drug initiation	
C2	**Drug form**<br>The cause of the DRP is related to the selection of the drug form	C2.1	Inappropriate drug form (for this patient)		Drug form
C3	**Dose selection**<br>The cause of the DRP can be related to the selection of the dosage schedule	C3.1	Drug dose too low		Dose selection
		C3.2	Drug dose too high		
		C3.3	Dosage regimen not frequent enough		
		C3.4	Dosage regimen too frequent		
		C3.5	Dose timing instructions wrong, unclear or missing		
C4	**Treatment duration**<br>The cause of the DRP is related to the duration of treatment	C4.1	Duration of treatment too short		Treatment duration
		C4.2	Duration of treatment too long		
C5	**Dispensing**<br>The cause of the DRP can be related to the logistics of the prescribing and dispensing process	C5.1	Prescribed drug not available		Dispensing
		C5.2	Necessary information not provided		
		C5.3	Wrong drug, strength or dosage advised (OTC)		
		C5.4	Wrong drug or strength dispensed		
C6	**Drug use process**<br>The cause of the DRP is related to the way the patient gets the drug administered by a health professional or carer, in spite of proper	C6.1	Inappropriate timing of administration or dosing intervals		Drug use process
		C6.2	Drug under-administered		
		C6.3	Drug over-administered		
		C6.4	Drug not administered at all		
		C6.5	Wrong drug administered		

Code	Domains	Code	Problem/ Cause/ Intervention		
			Original version (V9.0)	Modified version for intervention	Modified version for consultation
	instructions (on the label)	C6.6	Drug administered via wrong route		
C7 #	**Patient related**	**C7.1**	Patient uses/takes less drug than prescribed or does not take the drug at all		**Drug-related adverse effects or drug-induced disease**
	The cause of the DRP can be related to the patient and his behavior (intentional or non-intentional)	**C7.2**	Patient uses/takes more drug than prescribed		
		**C7.3**	Patient abuses drug (unregulated overuse)		
		**C7.4**	Patient uses unnecessary drug		
		**C7.5**	Patient takes food that interacts		
		**C7.6**	Patient stores drug inappropriately		
		**C7.7**	Inappropriate timing or dosing intervals		
		**C7.8**	Patient administers/uses the drug in a wrong way		
		**C7.9**	Patient unable to use drug/form as directed		
		**C7.10**	Patient unable to understand instructions properly		
C8 #	**Patient transfer related**	**C8.1**	No medication reconciliation at patient transfer		**Others, such as drug information**
	The cause of the DRP can be related to the transfer of patients between primary, secondary and tertiary care, or transfer within one care institution.	**C8.2**	No updated medication list available		
		**C8.3**	Discharge/transfer information about medication incomplete or missing		
		**C8.4**	Insufficient clinical information about the patient		
		**C8.5**	Patient has not received necessary medication at discharge from hospital or clinic		
**C9**	**Other**	**C9.1**	No or inappropriate outcome monitoring (incl. TDM)		/
		**C9.2**	Other cause; specify		
		**C9.3**	No obvious cause		
Intervention					
**I0**	**No intervention**	**I0.1**	No intervention		/
**I1**	**At prescriber level**	**I1.1**	Prescriber informed only		

Code	Domains	Code	Problem/ Cause/ Intervention		
			**Original version (V9.0)**	**Modified version for intervention**	**Modified version for consultation**
		**I1.2**	Prescriber asked for information		
		**I1.3**	Intervention proposed to prescriber		
		**I1.4**	Intervention discussed with prescriber		
**I2**	**At patient level**	**I2.1**	Patient (drug) counselling		
		**I2.2**	Written information provided (only)		
		**I2.3**	Patient referred to prescriber		
		**I2.4**	Spoken to family member/caregiver		
**I3**	**At drug level**	**I3.1**	Drug changed to …		
		**I3.2**	Dosage changed to …		
		**I3.3**	Formulation changed to …		
		**I3.4**	Instructions for use changed to …		
		**I3.5**	Drug paused or stopped		
		**I3.6**	Drug started		
		**I3.7 ***	/	**Needs adjustment on parenteral nutrition**	
		**I3.8 ***	/	**Necessary frequency adjustment needed based on antibiotics' PK/PD characteristics**	
**I4**	**Other intervention or activity**	**I4.1**	Other intervention (specify)		
		**I4.2**	Side effect reported to authorities		

* Being modified in intervention problems classification

Being modified in consultation problems classification

Supplementary Table 2. Top3 Medicines' Drug Related Problems and Causes of Pharmacy Intervention

Problem	Cause	N
Antibiotic		286
P1.1 No effect of drug treatment	C1.1 Inappropriate drug according to guidelines/formulary	10
	C3.2 Drug dose too high	1
	C9.1 No or inappropriate outcome monitoring (incl. TDM)	1
P1.2 Effect of drug treatment not optimal	C1.1 Inappropriate drug according to guidelines/formulary	16
	C1.3 No indication for drug	1
	C1.6 No or incomplete drug treatment in spite of existing indication	7
	C1.7 Too many drugs prescribed for indication	1
	C1.8 Necessary genetic testing before drug initiation	3
	C3.1 Drug dose too low	33
	C3.2 Drug dose too high	1
	C3.3 Dosage regimen not frequent enough	30
	C3.4 Dosage regimen too frequent	1
	C6.1 Inappropriate timing of administration or dosing intervals	1
	C9.1 No or inappropriate outcome monitoring (incl. TDM)	4
P1.3 Untreated symptoms or indication	C1.6 No or incomplete drug treatment in spite of existing indication	8
	C9.1 No or inappropriate outcome monitoring (incl. TDM)	1
P2.1 Adverse drug event (possibly) occurring	C1.1 Inappropriate drug according to guidelines/formulary	3
	C1.2 Inappropriate drug (within guidelines but otherwise contraindicated)	12
	C1.4 Inappropriate combination of drugs, or drugs and herbal medications, or drugs and dietary supplements	1
	C1.5 Inappropriate duplication of therapeutic group or active ingredient	2
	C1.6 No or incomplete drug treatment in spite of existing indication	1
	C1.7 Too many drugs prescribed for indication	1
	C1.8 Necessary genetic testing before drug initiation	2
	C3.2 Drug dose too high	37
	C4.2 Duration of treatment too long	4
	C9.1 No or inappropriate outcome monitoring (incl. TDM)	11
P3.2 Unnecessary drug-treatment	C1.1 Inappropriate drug according to guidelines/formulary	2
	C1.2 Inappropriate drug (within guidelines but otherwise contraindicated)	1
	C1.3 No indication for drug	8
	C1.5 Inappropriate duplication of therapeutic group or active ingredient	6
	C1.7 Too many drugs prescribed for indication	1
	C3.2 Drug dose too high	2
	C4.2 Duration of treatment too long	27
	C9.1 No or inappropriate outcome monitoring (incl. TDM)	1
P3.3 Needs Supplementary drug therapeutic monitoring	C1.2 Inappropriate drug (within guidelines but otherwise contraindicated)	1
	C1.8 Necessary genetic testing before drug initiation	4
	C3.2 Drug dose too high	1

Problem	Cause	N
(TDM)	C4.2 Duration of treatment too long	1
	C9.1 No or inappropriate outcome monitoring (incl. TDM)	32
P3.4 Antibiotics De-escalation	C1.1 Inappropriate drug according to guidelines/formulary	5
	C4.2 Duration of treatment too long	1
Parenteral nutrition		28
P1.2 Effect of drug treatment not optimal	C1.6 No or incomplete drug treatment in spite of existing indication	4
	C3.2 Drug dose too high	1
P1.3 Untreated symptoms or indication	C1.6 No or incomplete drug treatment in spite of existing indication	3
P2.1 Adverse drug event (possibly) occurring	C1.1 Inappropriate drug according to guidelines/formulary	1
	C1.2 Inappropriate drug (within guidelines but otherwise contraindicated)	4
	C1.5 Inappropriate duplication of therapeutic group or active ingredient	1
	C1.7 Too many drugs prescribed for indication	1
	C3.1 Drug dose too low	2
	C3.2 Drug dose too high	2
P3.1 Problem with cost-effectiveness of the treatment	C1.3 No indication for drug	1
	C2.1 Inappropriate drug form (for this patient)	1
P3.2 Unnecessary drug-treatment	C1.3 No indication for drug	5
	C4.2 Duration of treatment too long	2
Proton pump inhibitor		13
P2.1 Adverse drug event (possibly) occurring	C1.6 No or incomplete drug treatment in spite of existing indication	1
	C3.2 Drug dose too high	3
P3.2 Unnecessary drug-treatment	C4.2 Duration of treatment too long	9

Supplementary Table 3. Top3 Medicines' Drug Related Problems and Causes of Pharmacy Consultation

Problem	Cause	N
Antibiotic		153
P1.1 No effect of drug treatment	C1 Drug selection	1
	C3 Dose selection	2
P1.2 Effect of drug treatment not optimal	C1 Drug selection	6
	C3 Dose selection	10
P1.3 Untreated symptoms or indication	C1 Drug selection	7
	C3 Dose selection	12
P1.4 Other	C3 Dose selection	1
P2.1 Adverse drug event (possibly) occurring	C1 Drug selection	6
	C3 Dose selection	78
	C6 Drug use process	3
	C7 Drug-related side effects/Drug-induced diseases	9
	C8 Other	3
P3 Treatment safety and cost-effectiveness	C1 Drug selection	1
	C3 Dose selection	10
	C4 Treatment duration	1
P4 Other	C3 Dose selection	1
	C4 Treatment duration	1
	C6 Drug use process	1
Antifungal drugs		21
P1.2 Effect of drug treatment not optimal	C3 Dose selection	1
P1.3 Untreated symptoms or indication	C1 Drug selection	3
	C3 Dose selection	4
	C8 Other	1
P1.4 Other	C4 Treatment duration	1
P2.1 Adverse drug event (possibly) occurring	C1 Drug selection	2
	C3 Dose selection	4
	C7 Drug-related side effects/Drug-induced diseases	3
P4 Other	C3 Dose selection	1
	C8 Other	1
Antiepileptic drugs		9
P1.2 Effect of drug treatment not optimal	C3 Dose selection	3
P1.3 Untreated symptoms or indication	C3 Dose selection	1
P2.1 Adverse drug event (possibly) occurring	C3 Dose selection	2
P4 Other	C3 Dose selection	1
	C8 Other	2
