# Supplementary material for: Drug-Related Problems Identified During Pharmacy Intervention and Consultation: Implementation of an Intensive Care Unit Pharmaceutical Care Model
Source: Front Pharmacol. 2020 Sep 11;11:571906. doi: 10.3389/fphar.2020.571906 (PMC7516263; doi:10.3389/fphar.2020.571906)
Supplement: Supplementary file 2 [file Presentation_2.pdf]

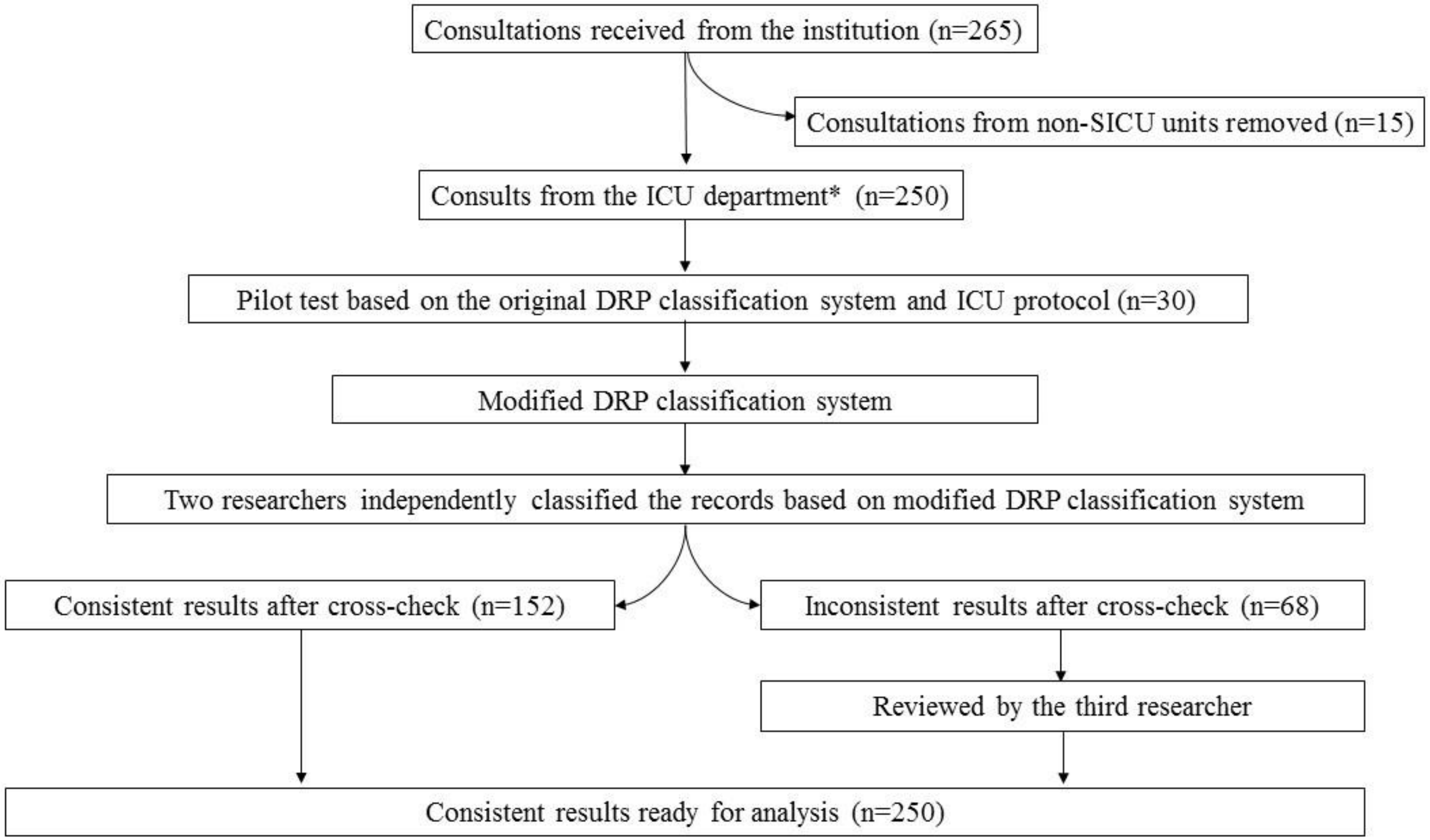

Supplementary Figure 1. The flowchart for consultation classification * Including 8 consults received at the pharmacy department level.

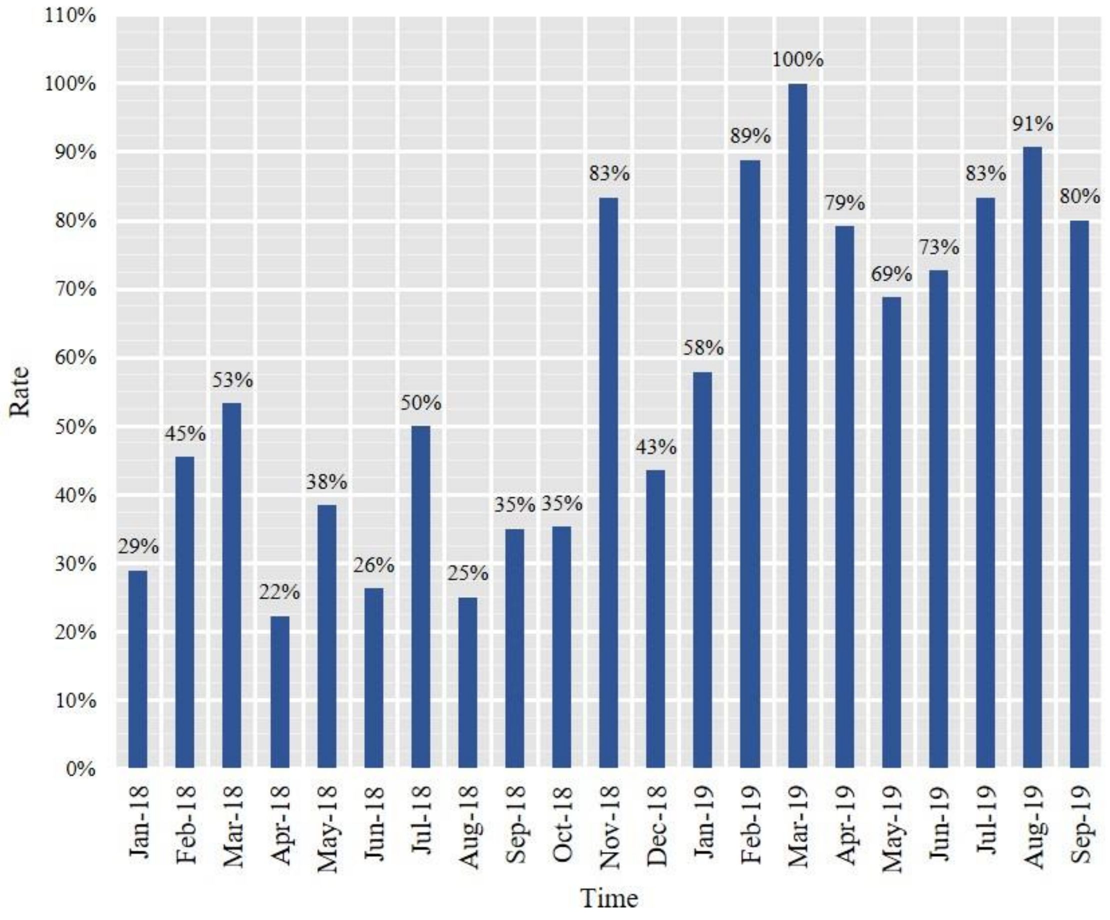

Supplementary Figure 2. Monthly Intervention Rate during Pharmacy intervention Monthly

The monthly intervention rate (%) = $\frac{The\ number\ of\ intervention\ patients}{The\ number\ of\ close\ monitoring\ patients} * 100\%$.

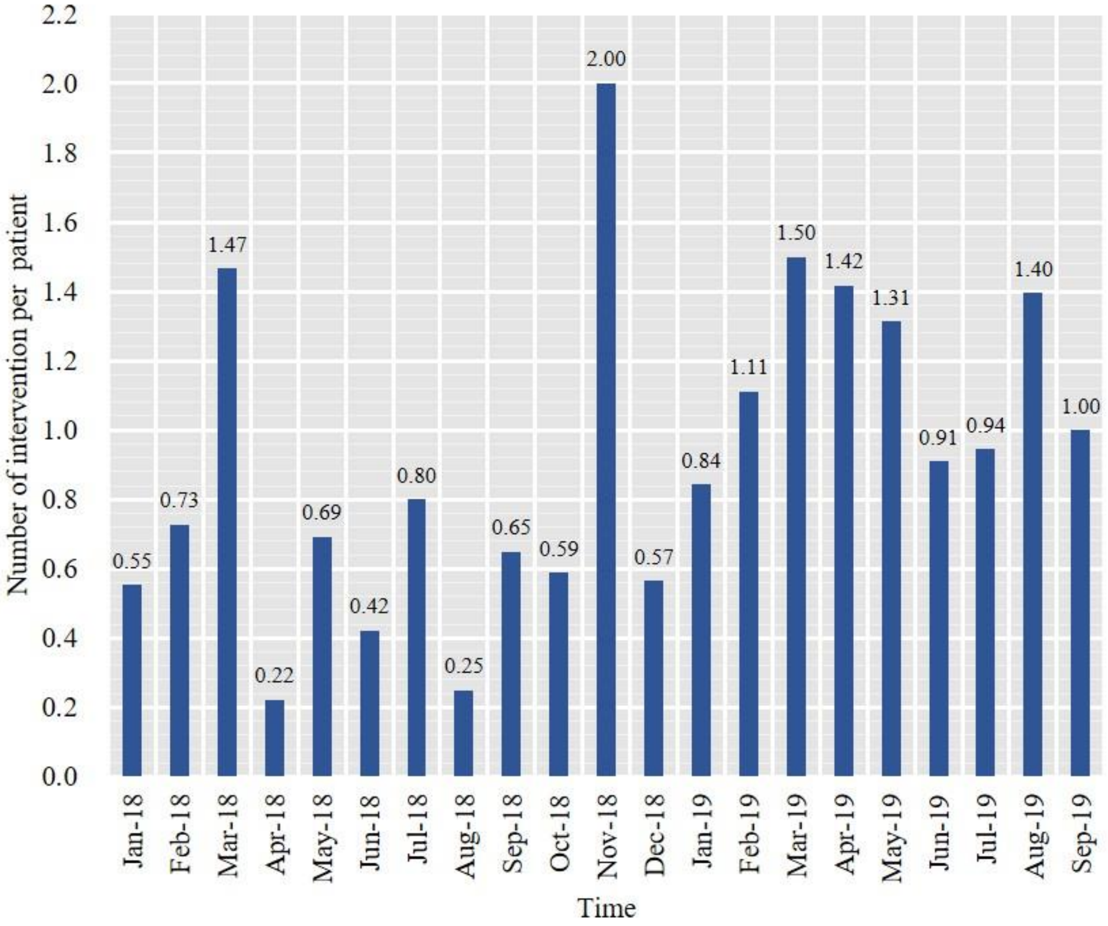

Supplementary Figure 3. Monthly Number of Intervention per Patient during Pharmacy intervention The monthly number of intervention per patient= $\frac{The\ number\ of\ intervention\ cases}{The\ number\ of\ close\ monitoring\ patients}$.

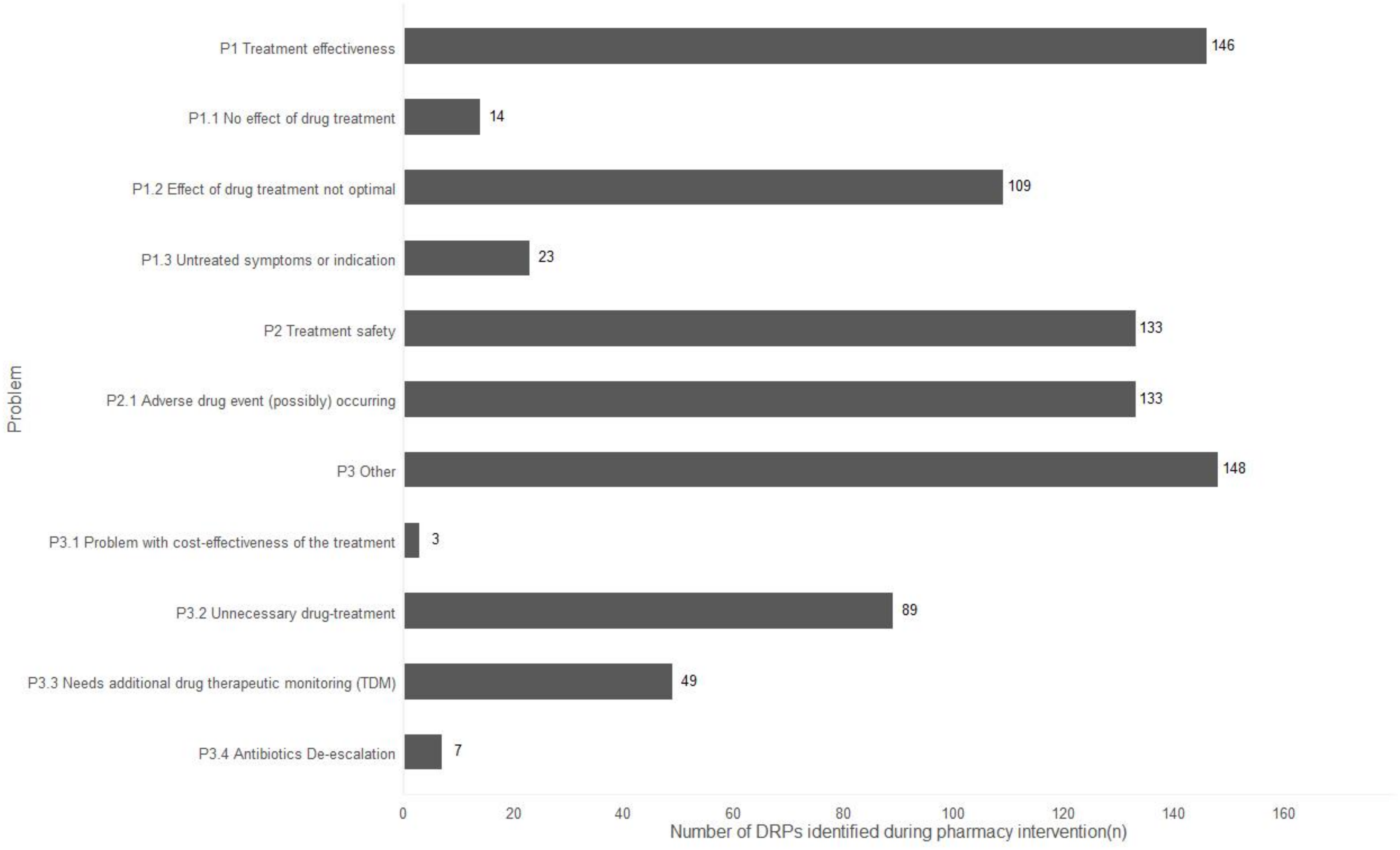

Supplementary Figure 4. Number of DRPs Identified during Pharmacy intervention

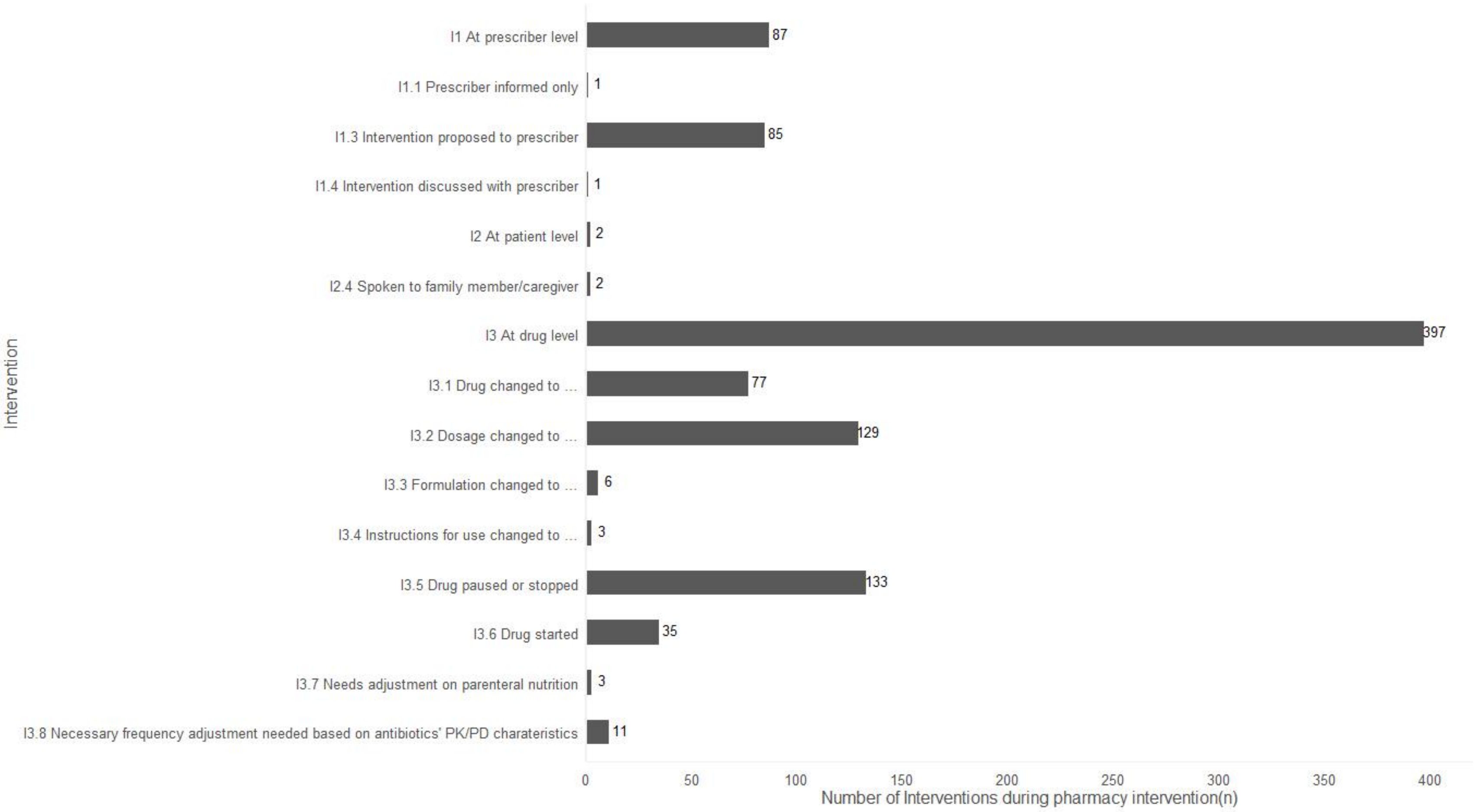

Supplementary Figure 5. Number of Intervention during Pharmacy intervention

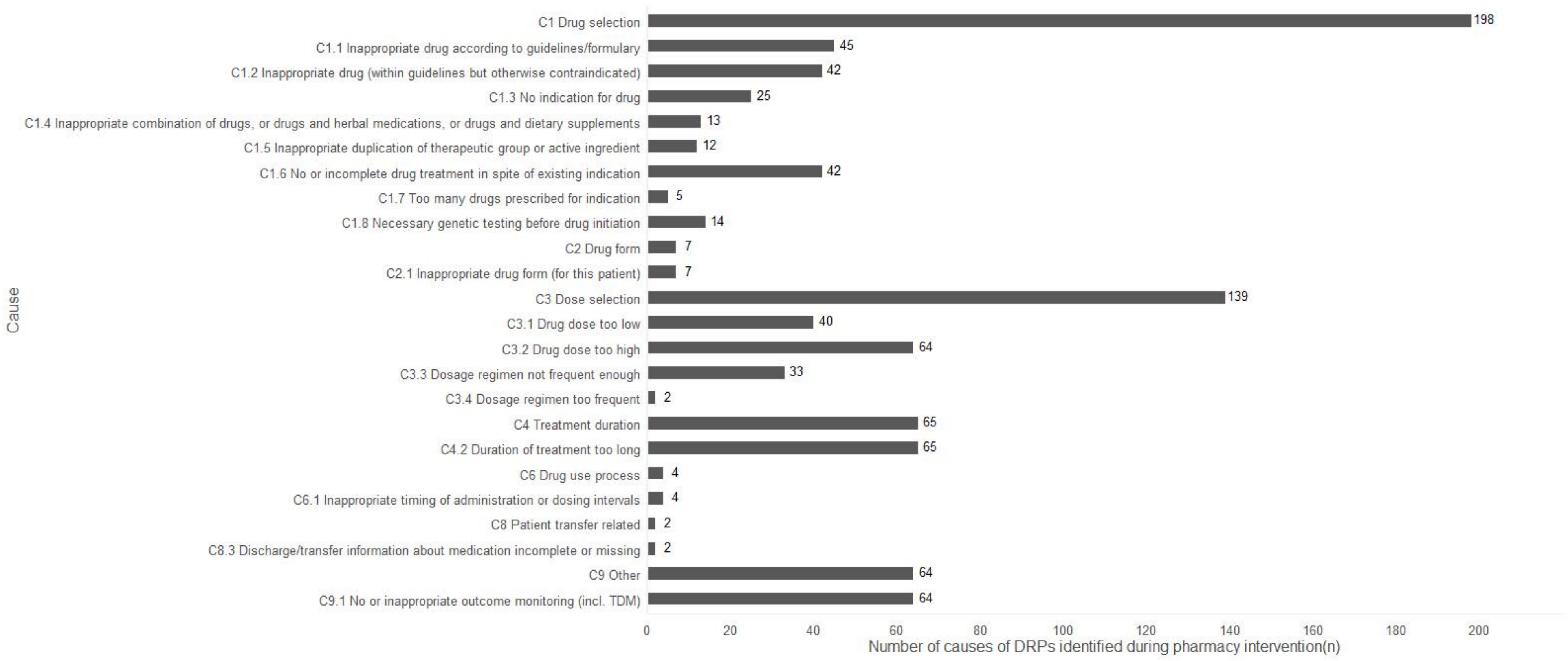

Supplementary Figure 6. Number of Causes of DRPs Identified during Pharmacy Intervention

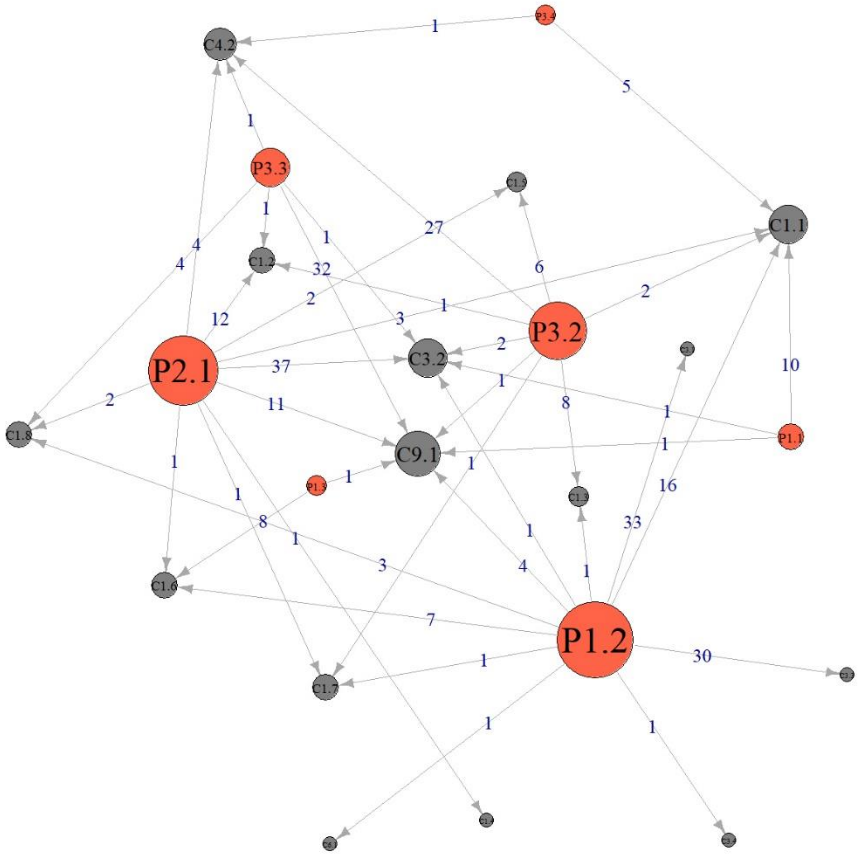

Supplementary Figure 7. Network of Causes and Correspond DRPs of Antibiotic Medicines Identified During Pharmacy Intervention A network graph was used to describe the relationship between the DRPs and Causes of antibiotics identified during pharmacy intervention. The size of the circle indicates how many times this DRPs or Cause was identified. An arrow pointing from P (DRPs) to C (Causes) means the problem is caused by the corresponding cause, and the number on the line indicates the frequency of this causal relationship. The main Cause leading to P2.1 Adverse drug event (possibly) occurring was C3.2 Drug dose too high (n=37), followed by C1.2 Inappropriate drug (within guidelines but otherwise contraindicated) (n=12) and C9.1 No or inappropriate outcome monitoring (n=11), and the main Cause leading to P1.2 Effect of drug treatment not optimal was C3.1 Drug dose too low (n=33), followed by C3.3 Dosage regimen not frequent enough (n=30), C1.1 Inappropriate drug according to guidelines/formulary (n=16) and C1.6 No or incomplete drug treatment in spite of existing indication (n=7). P3.2 Unnecessary drug-treatment was mainly caused by C1.3 No indication for drug (n=8), and P3.3 Dosage regimen not frequent enough by C9.1 No or inappropriate outcome monitoring (incl. TDM) (n=32).

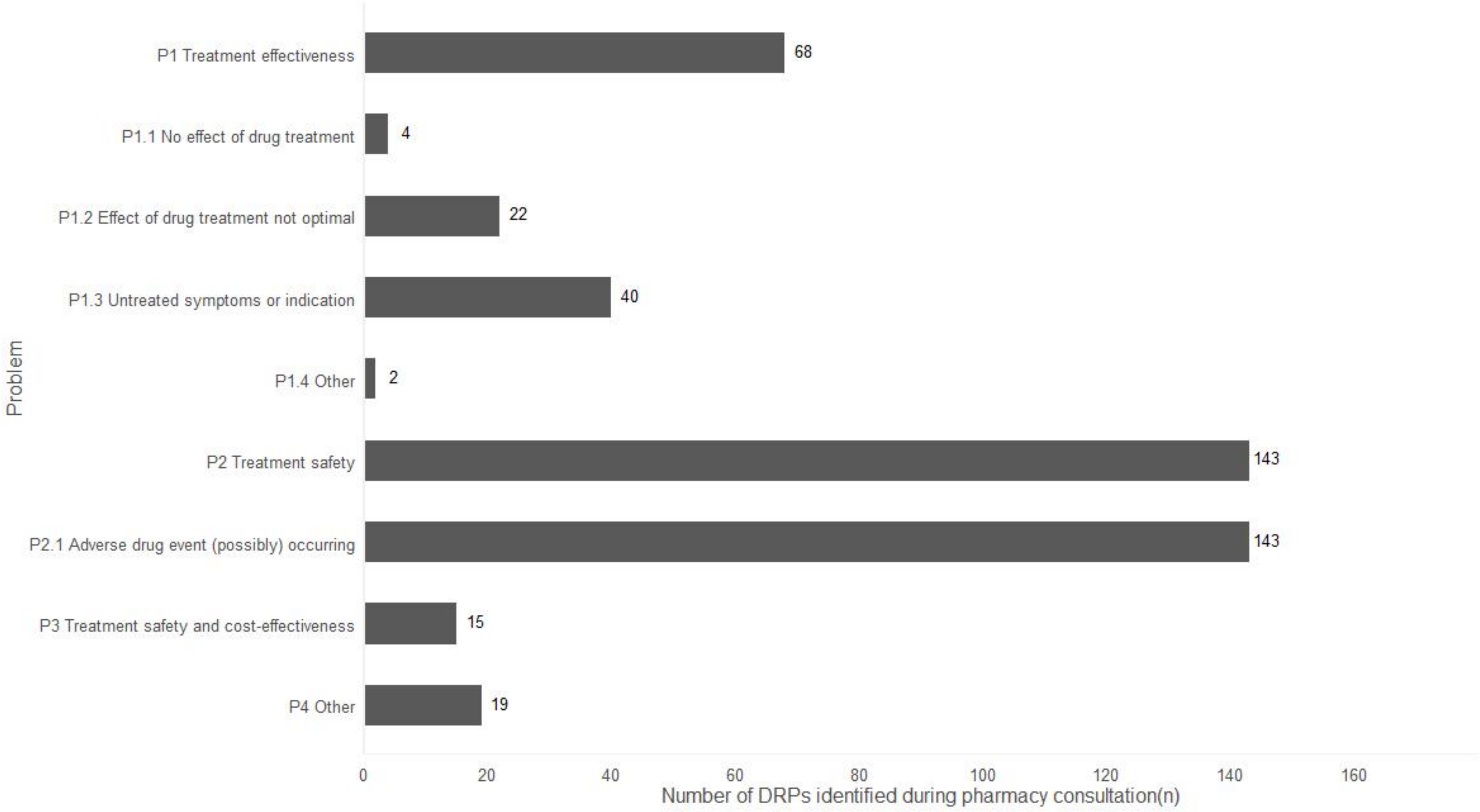

Supplementary Figure 8. Number of DRPs Identified during Pharmacy Consultation

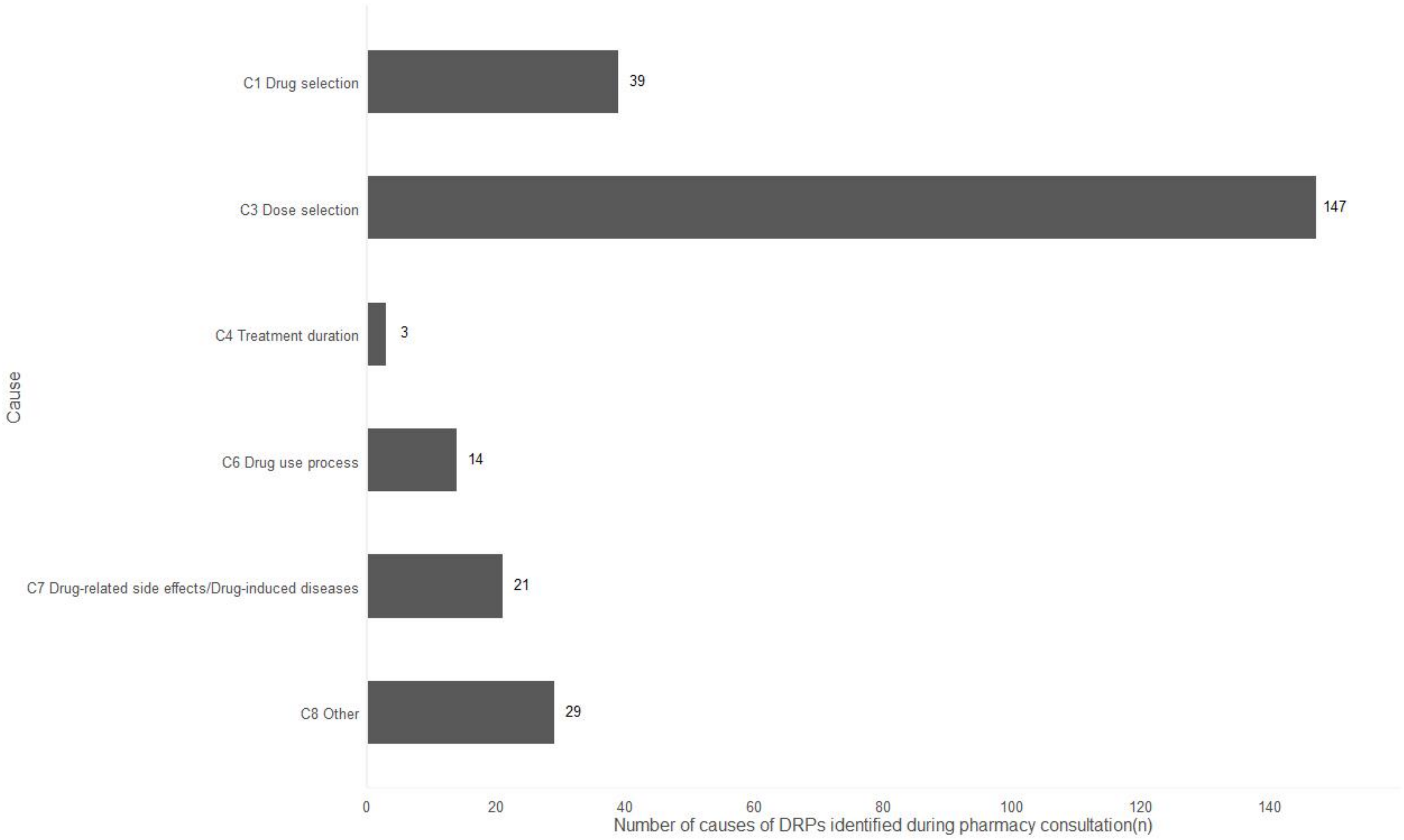

Supplementary Figure 9. Number of Causes of DRPs Identified during Pharmacy Consultation

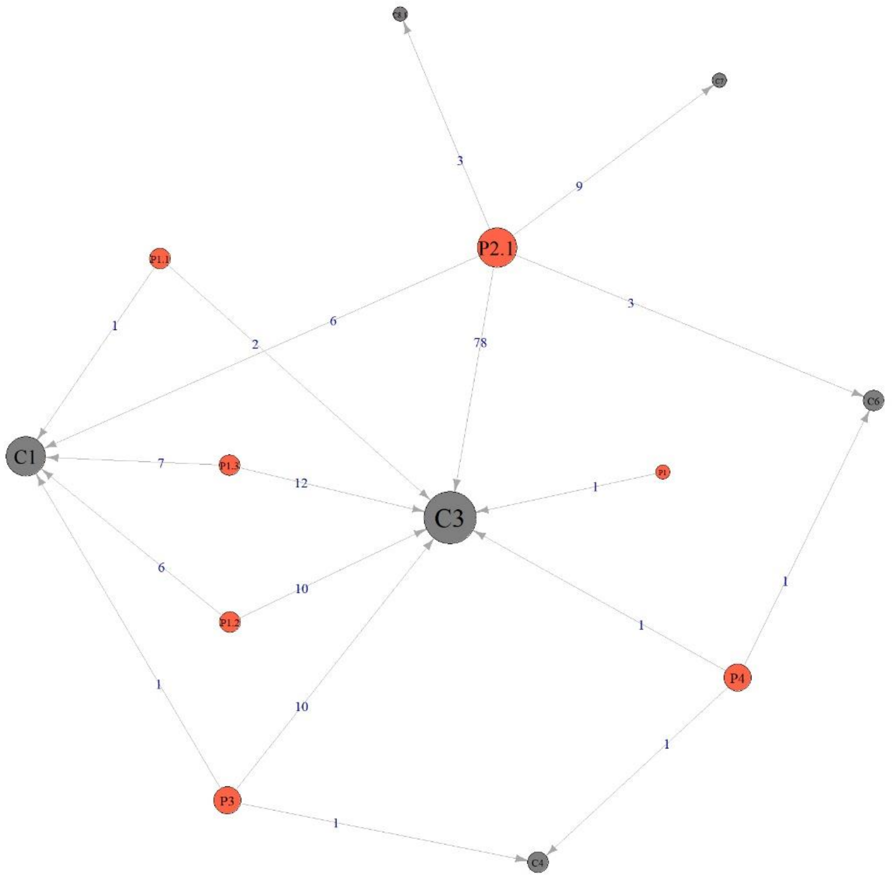

Supplementary Figure 10. Network of Causes and Corresponding DRPs of Antibiotic Medicines Identified during Pharmacy Consultation A network graph was used to describe the relationship between the DRPs and Causes identified during pharmacy intervention. The size of the circle indicates how many times this DRP or Cause was identified. An arrow pointing from P (DRP) to C (Cause) means the problem is caused by the corresponding cause, and the number on the line indicates the frequency of this causal relationship. The main Cause leading to P2.1 Adverse drug event (possibly) occurring was C3 Dose selection (n=94), followed by C7 Drug-related adverse effects or drug-induced disease (n=18) and C1 Drug/Drug regimen selection (n=13), and the main Cause leading to P1.3 Untreated symptoms or indication was C3 Dose selection (n=20) and C1 Drug/Drug regimen selection (n=16).
